# A Multi-Area Stochastic Model for a Covert Visual Search Task

**DOI:** 10.1371/journal.pone.0136097

**Published:** 2015-08-19

**Authors:** Michael A. Schwemmer, Samuel F. Feng, Philip J. Holmes, Jacqueline Gottlieb, Jonathan D. Cohen

**Affiliations:** 1 Mathematical Biosciences Institute, The Ohio State University, Columbus, OH 43210, United States of America; 2 Department of Applied Mathematics and Sciences, Khalifa University, Abu Dhabi, United Arab Emirates; 3 Program in Applied and Computational Mathematics, Department of Mechanical and Aerospace Engineering, and Princeton Neuroscience Institute, Princeton University, Princeton, NJ 08544, United States of America; 4 Department of Neuroscience, Columbia University, New York, NY 10032, United States of America; 5 Department of Psychology and Princeton Neuroscience Institute, Princeton University, Princeton, NJ 08544, United States of America; Centre de Neuroscience Cognitive, FRANCE

## Abstract

Decisions typically comprise several elements. For example, attention must be directed towards specific objects, their identities recognized, and a choice made among alternatives. Pairs of competing accumulators and drift-diffusion processes provide good models of evidence integration in two-alternative perceptual choices, but more complex tasks requiring the coordination of attention and decision making involve multistage processing and multiple brain areas. Here we consider a task in which a target is located among distractors and its identity reported by lever release. The data comprise reaction times, accuracies, and single unit recordings from two monkeys’ lateral interparietal area (LIP) neurons. LIP firing rates distinguish between targets and distractors, exhibit stimulus set size effects, and show response-hemifield congruence effects. These data motivate our model, which uses coupled sets of leaky competing accumulators to represent processes hypothesized to occur in feature-selective areas and limb motor and pre-motor areas, together with the visual selection process occurring in LIP. Model simulations capture the electrophysiological and behavioral data, and fitted parameters suggest that different connection weights between LIP and the other cortical areas may account for the observed behavioral differences between the animals.

## Introduction

Decisions pervade our daily lives. They typically involve the coordination of several steps, including attending to relevant stimuli, extracting the evidence therein, and selecting an appropriate action. Significant advances in understanding the neural mechanisms of decision making have come from studies of the monkey oculomotor system, which have tested the neural correlates of simple perceptual decisions. For instance, when discriminating motion direction in a random dot kinematogram (e.g. [1, 2]). neurons in the lateral intraparietal area (LIP), exhibit spatially-tuned visuo-motor responses that signal the action with which monkeys report their decisions: a saccade inside or opposite their visual receptive field (RF) [3–6]. Decision-related LIP firing rates (FRs) can be fit remarkably well by pairs of leaky competing accumulators [7], which can in turn be reduced to drift-diffusion processes [8]. Such models are remarkably successful in capturing reaction time distributions and error rates, providing compelling motivation for their continued use [9, 10]

In these investigations the relevant sensory evidence is clear, but in nature animals are surrounded by multiple stimuli and, in addition to deciding which action to take, must decide which stimulus to attend to. Thus, natural decisions entail at least two interdependent processes: selection of a relevant source of information and selection of an appropriate course of action. Most previous modeling studies have considered the neural correlates of attention or action selection, but not their interactions (exceptions include [11, 12]). More abstract connectionist networks have addressed interactions among brain areas supporting different processes (e.g., feature representation vs. location-specific information [13]), but they have generally lacked sufficient detail to make contact with neurophysiological data. Here, we present a model that attempts to bridge this gap, based on a task in which LIP neurons encode interacting effects of visual and motor selection.

**Fig 1:**
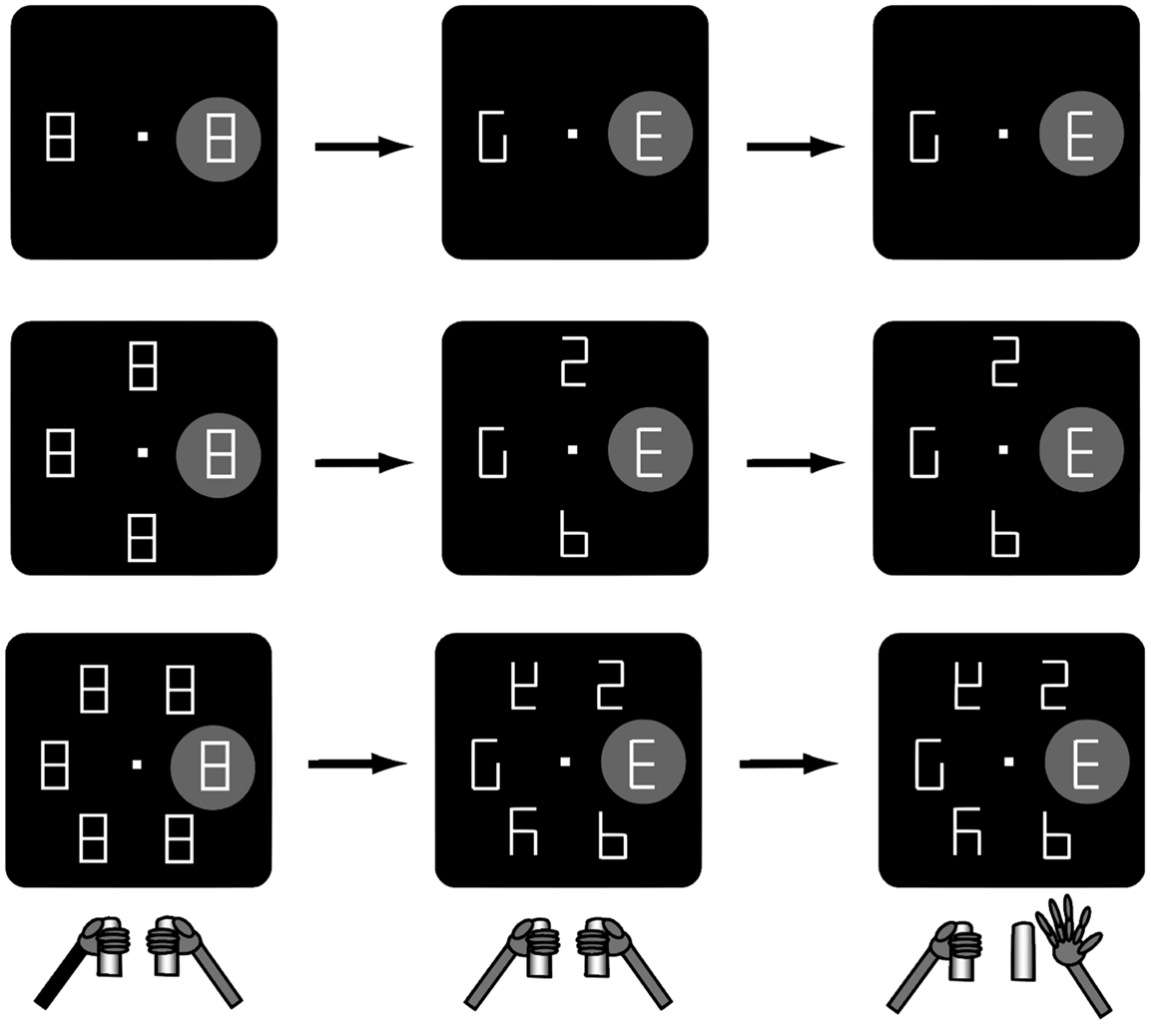
The covert search task. Displays of set sizes 2, 4 and 6 are shown top to bottom. Monkeys initiated a trial by fixating on a central point and grasping two bars; they were required to maintain fixation throughout the trial. One figure eight placeholder always fell in the recorded neuron’s receptive field (RF, gray disc). After 500 msec, two line segments were removed from each figure, revealing distractors and one target: a left- or right-facing 𝖤 (latter shown here). Correct responses, signalled by respective release of left (L) or right (R) bar were rewarded. Figure from [15] according to Creative Commons Attribution License.

In the behavioral paradigm (shown below in Fig 1), monkeys were required to discriminate whether a target (an E-like shape) was oriented to the right or left, and indicate their responses by releasing a bar that was held, respectively, in the right or left paw [14, 15]. Therefore, as in earlier paradigms, the monkeys made a binary perceptual decision and signaled it with a motor action. However, in contrast with those studies, the target was surrounded by irrelevant distractors and, before responding, the monkeys had to use covert attention to find the target in the distractor array. LIP neurons encoded correlates of both the visual and the motor selections. The neurons responded with higher FRs if the target rather than a distractor was in their receptive field (RF), signaling the visual selection, and this visuo-spatial response was sensitive to the motor action, being stronger or weaker when the monkeys released the right or the left paw. This complex coupling of attention and decision-related responses suggests that pairs of competing accumulators that describe only the final decision alternatives (i.e., the two manual actions) may not adequately capture the decision process or the role of LIP within it.

Here we construct a model containing several accumulators that implement visual and motor selection and interact during decision making. To focus our model, we do not include processes related to early visual representation or target search and discrimination, but focus on the 3-way interactions between a shape selective representation, a visual selection area and a motor selection system. While the properties of the shape selective and motor stages are inferred from the literature, the visual selection stage is modeled based on the LIP data. The model produces acceptable fits not only of LIP neural responses but also reaction time (RT) distributions and accuracy, and captures individual differences between two monkeys through changes in connection strengths among the different modules. Thus we show that a framework built on the competing accumulator mechanism that has been applied to simple perceptual decisions can be extended to account for more complex decisions that involve interaction among distinct processes of visual and skeleto-motor selection.

## Analyses and Methods

**Fig 2:**
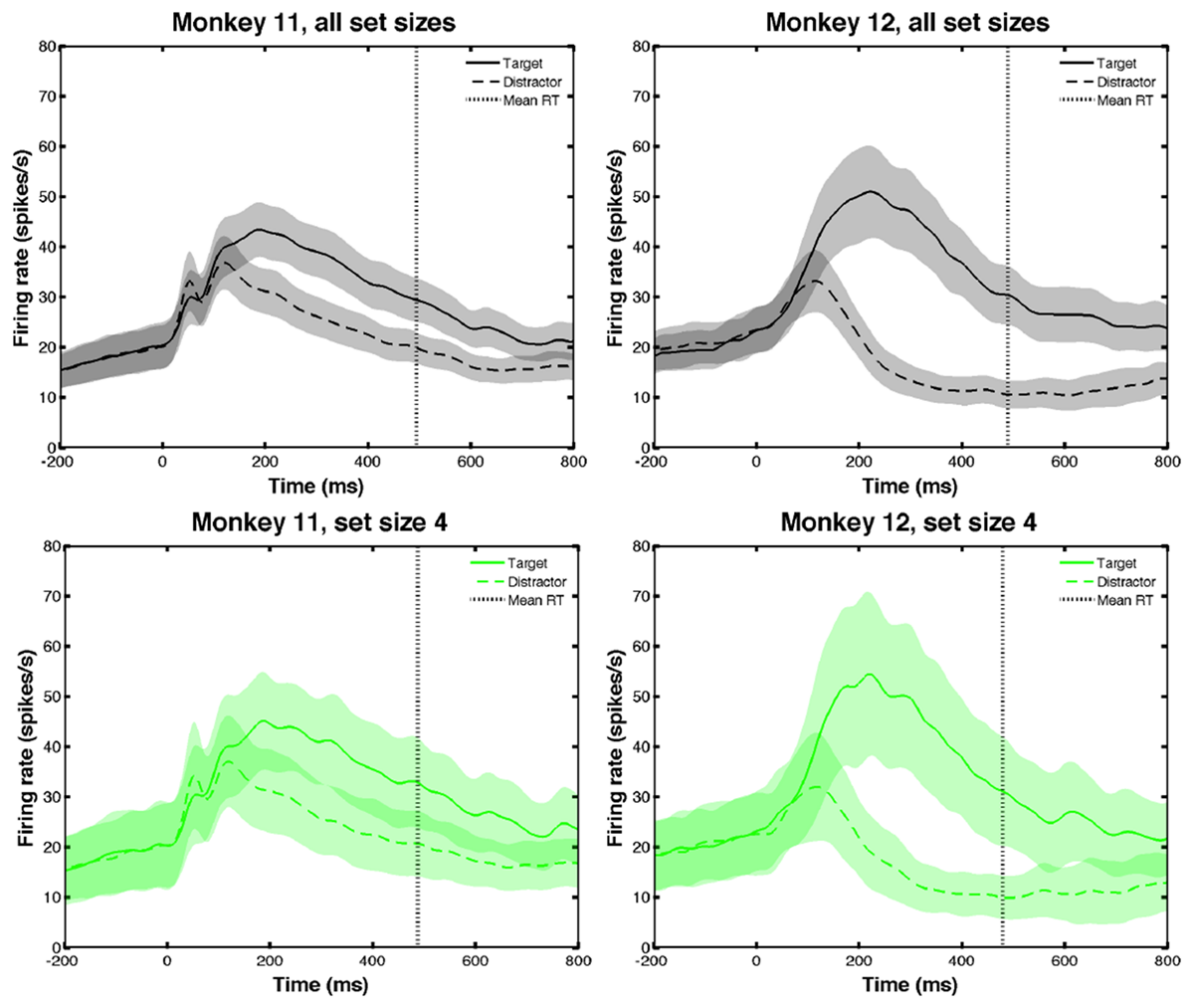
LIP neurons show differences in activity for targets and distractors in their receptive fields. Mean firing rates (FRs) in LIP in M11 (left) and M12 (right) for target (solid) and distractor (dashed) in receptive field (RF) respectively; correct and incorrect trials are included. Top panels show averages over all set sizes. Cue onset occurs at *t* = 0 and vertical dotted black lines represent overall mean reaction times (RTs) (486.9 ms, M11 and 482.9 ms, M12); shaded regions represent one standard error from the mean (SEM). Bottom panels show averages over set size 4 data; see Table 1 for mean RTs for each set size.

**Fig 3:**
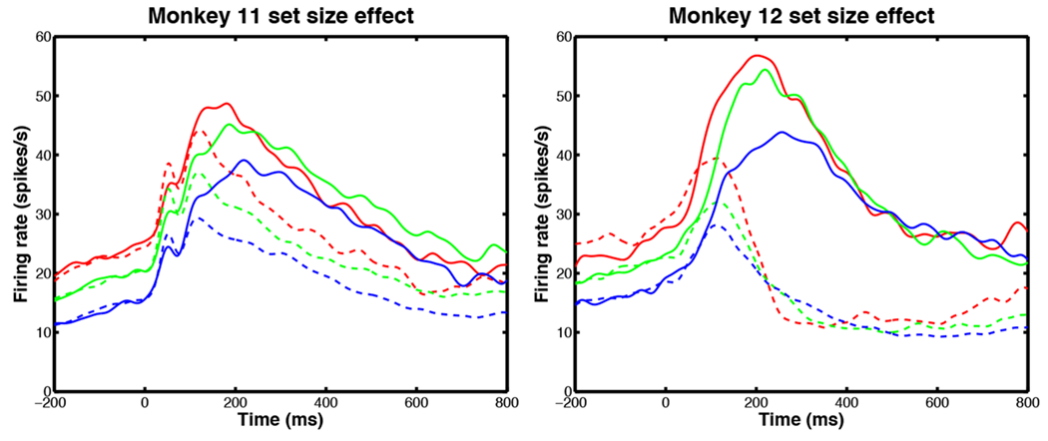
LIP neurons display differing levels of activity depending upon the number of visual objects on screen. Mean firing rates (FRs) in LIP in monkey 11 (M11, left) and monkey 12 (M12, right) for different set sizes, averaged over all neurons for which all three set sizes were tested. As set size increased from 2 (red) to 4 (green) to 6 (blue), firing rates decreased for both target (solid) and distractor (dashed) in receptive field (RF). Standard errors are not shown to avoid clutter.

**Fig 4:**
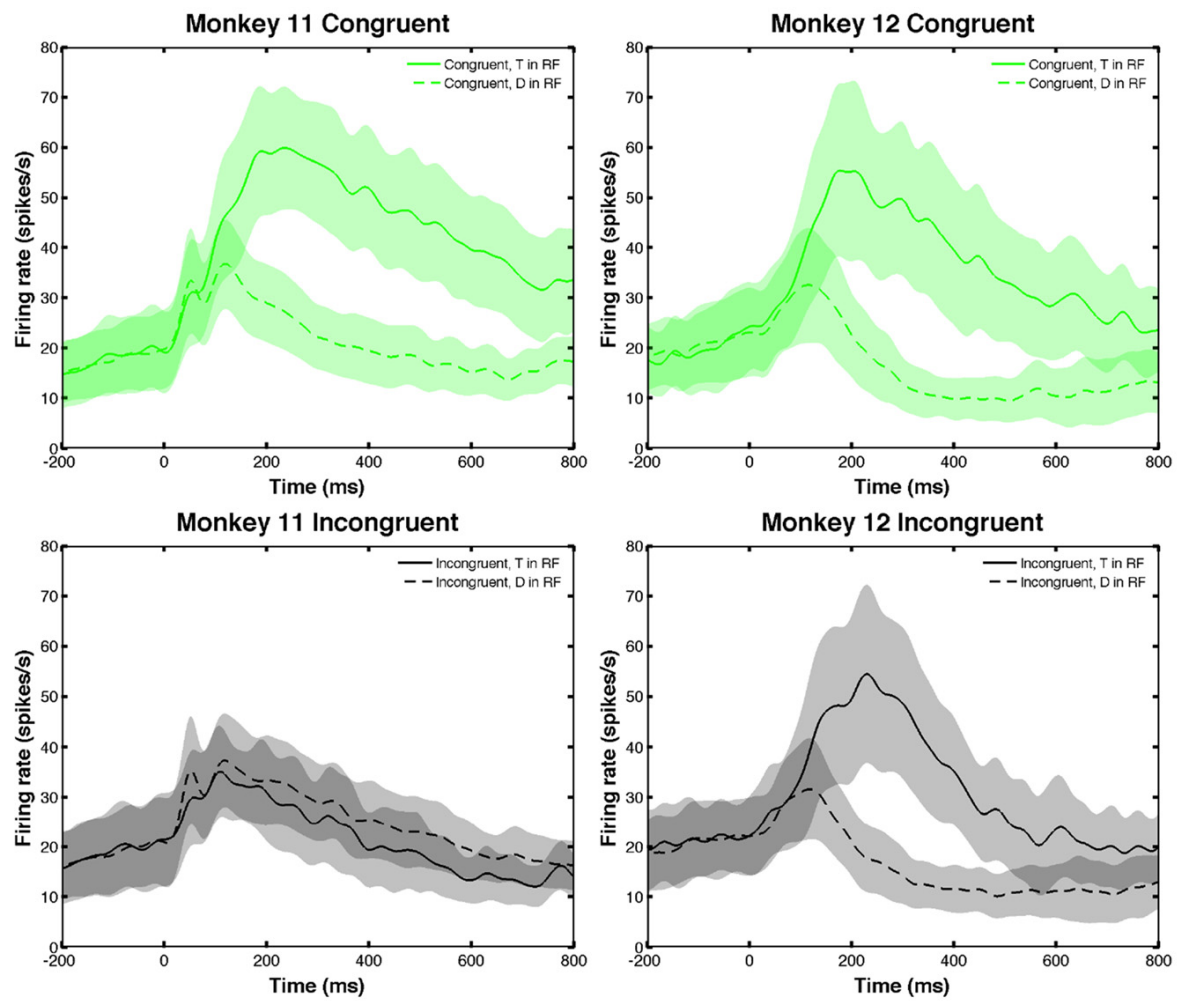
LIP neurons in one monkey display differing levels of activity depending upon the orientation of the target and its position in the left or right visual hemifield. Response-hemifield congruence effects for set size 4. M11 (left) exhibited significantly greater firing rates (FRs) for congruent (upper, green) versus incongruent (lower, black) trials with target in receptive field (RF) (solid) (*p* = 1.723 × 10^−49^); FR differences for distractor in RF (dashed) were not significant (*p* = 0.05649). M12 (right) showed no significant difference in FRs for congruent versus incongruent trials with either target or distractor in RF (*p* = 0.09932 and *p* = 0.2472 respectively). Shaded regions represent one SEM.

**Fig 5:**
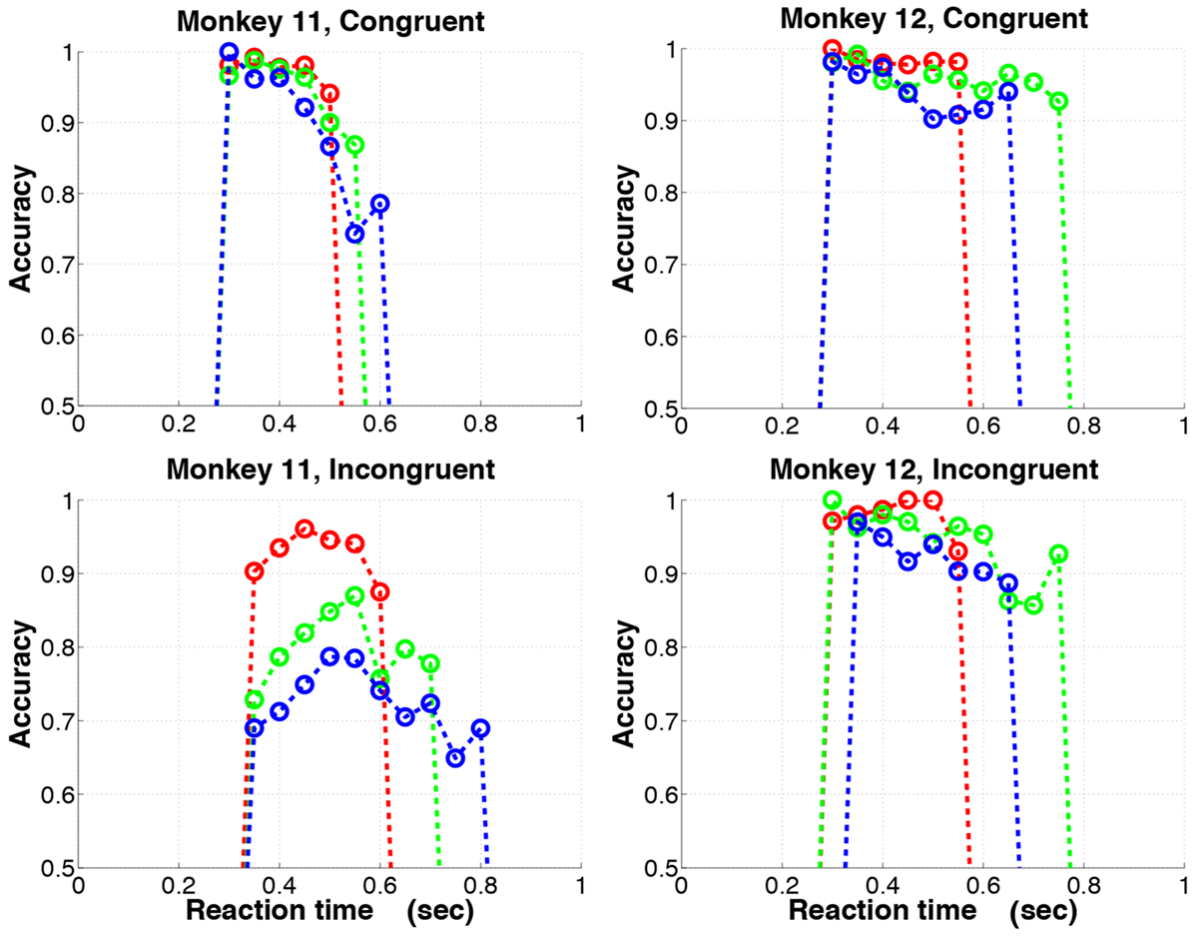
Response-hemifield congruence also affects accuracy and reaction time. Accuracy as a function of reaction time (RT) for set sizes 2 (red), 4 (green), and 6 (blue). Downward trends in accuracy at longer RTs are greater in M11 than M12, and more marked for incongruent trials. Set size effects are especially pronounced in M11 for incongruent trials.

Before developing the model, we review key observations from the electrophysiological and behavioral data published in [14, 15]. The re-analyses presented in the following sections and Figs 2–5 motivate and guide our model construction, after which we describe the methods used for fitting the model to the experimental data.

### The covert search task: key observations and data analysis

Two macaque monkeys (M11 and M12) performed a covert visual search task in which they discriminated the orientation of a single visual target or cue embedded in an array of stimuli, as shown in Fig 1 [14, 15]. To initiate a trial, a monkey fixated on the central point in a visual display with either 2, 4 or 6 figure eight placeholders, spaced equally around the periphery, the number being the *set size*. The monkey was also required to hold two bars located below the display, outside its visual field, one in each hand. After 500 msec, at *cue onset*, two line segments were removed from each placeholder so that one became the target and the others became distractors (having different forms for set sizes 4 and 6). The target was a letter 𝖤, oriented left (∃) or right (𝖤). Maintaining fixation throughout, monkeys had to find the target, identify its orientation and indicate their choice by releasing either the left (L) or right (R) bar with the corresponding limb.

Single unit spike time data was recorded from LIP neurons that had been identified using the memory saccade task [15]. After locating the neuron’s receptive field (RF), the display was oriented so that one placeholder lay entirely inside the recorded neuron’s RF. On each trial, the target location and orientation (𝖤, ∃) were varied uniformly at random across the 2, 4, or 6 locations. Trials were administered in randomly-interleaved blocks with set size fixed throughout each block, and RTs and accuracy were logged. Blocks were typically arranged into sessions in which recordings were made from the same cell, while set size changed across blocks. At most one session of data was collected each day from one of two monkeys (M11 and M12). For M11 there were 46 sessions containing 108 blocks, totaling 11768 trials. For M12 there were 61 sessions containing 124 blocks, totaling 9661 trials. Details of methods and equipment used can be found in [14, 15]. Also, the recorded data is available as S1 Datasets.

The results in this section, including some that previously appeared in [14, 15], were obtained by reanalyzing the original behavioral and spike train data in preparation for building and fitting the model we discuss below. FRs were computed by convolving spike times with a Gaussian distribution of standard deviation 15 msec and averaging over appropriate trials, aligned at cue onset. To reproduce FRs reported in [15], we excluded six cells with maximum FRs exceeding 80 Hz, substantially higher that all others (< 50 Hz): cells 96, 154, and 210 for M11, and 196, 263, and 292 for M12. S1 Text lists the numbers of cells available for analysis after excluding these six. These numbers differ for each set size. In all analyses that follow and the data fits of our model, we include only those cells for which trials were available for all set sizes, and their associated behavioral data. There were 23 such cells for M11 and 21 for M12.

We now describe key features of the electrophysiological and behavioral data that our model incorporates. These include effects produced by target vs. distractor location in RF, set size effects, response-hemifield congruence, and accuracy versus RT patterns.

#### LIP neurons encode target location

In [14] it was found that the majority of LIP neurons encoded target location, responding strongly and selectively on trials in which the target fell in the neuron’s RF. Responses with distractors in the RF were much weaker, even though distractors had similar forms and brightnesses. Fig 2 shows these effects, locked to cue onset (i.e. *t* = 0 denotes the time at which two bars were removed from each figure eight). Shaded regions in the top panels of Fig 2 indicate one standard error from mean (SEM) FRs, averaged over all data; bottom panels show FRs of set size 4 data in each monkey.

**Table 1:** Accuracy and mean reaction time data for the two monkeys.

	Monkey 11		Monkey 12	
	Accuracy	Mean RT	Accuracy	Mean RT
**Set size 2**	95.5%	468.9±3.8	98.1%	462.0±5.5
**Set size 4**	88.5%	491.9±3.6	96.1%	489.9±4.6
**Set size 6**	83.1%	505.4±3.2	93.6%	503.2±4.9
**Congruent**	94.6%	448.9±2.2	95.9%	491.5±4.3
**Incongruent**	80.6%	535.3±3.0	95.4%	484.5±3.8

Accuracy and mean reaction times (RTs, in ms ± SEM) for different set sizes and response-hemifield congruency conditions. The top four rows show data for each set size, averaged over congruent and incongruent trials. The bottom two rows show data separately for congruent and incongruent trials, each averaged over all set sizes.

Fig 2 shows that steady increases in FR ensued after fixation (from −200 ms to shortly after cue onset). Thereafter both target and distractor FRs increased more rapidly for ≈ 100 ms, when distractor FRs peaked and began to decrease, while target FRs continued to increase for a futher ≈ 100 ms. Both FRs then decreased until after the response was made. Such encoding of behaviorally-relevant stimuli exemplify attentional effects that have previously been found in LIP [16]. Fig 2 also reveals differences between the two monkeys: M12 exhibits a higher peak FR than M11 when the target is in the neuron’s RF and M12’s FR decays more rapidly than M11’s with a distractor in the neuron’s RF. Also, differences between FRs with target and distractor in RF are greater for M12 than M11.

#### Neural and behavioral data exhibit set size effects

LIP activity and behavior were also affected by set size [15]. As the number of distractors increased, lower FRs were observed throughout the trial, as shown in Fig 3. This suppression occured with both target and distractors in the recorded neuron’s RF. Increases in set size also caused monkeys to respond more slowly and less accurately: an expected behavioral effect shown in Table 1. The set size effect was first demonstrated in [15], where it was noted that it might be due to long-range competitive interactions that limit neural activities in LIP related to spatial attention. Our model implements competition through the use of mutual inhibition among units representing different visual areas, and our model fitting results show that it can produce set size effects similar to those of Fig 3.

#### Neural and behavioral data show response-hemifield congruence effects

The covert search task has two stages: to select the informative target (in R or L hemifield) and then release the appropriate bar (with R or L hand). This leads to an interesting congruence effect, first reported in [14], and implicit in RTs shown in the bottom two rows of Table 1. A trial is called *response-hemifield congruent* if the correct L/R response required a bar release on the *same* side of the display as the target. Thus, congruent trials had right-facing 𝖤’s to the right of the fixation point and left-facing ∃’s to the left of the fixation point. Incongruent trials were those for which right-facing 𝖤’s were on the left and left-facing ∃’s were on the right, each requiring a lever release on the *opposite* side to that on which the target appeared (see [14, Fig 7). M11 responded much faster and more accurately on response-hemifield congruent versus incongruent trials. Surprisingly, M12 responded slightly slower on response-hemifield congruent trials, with similar accuracy on both trial types. Furthermore, when the target was in the RF, LIP FRs differed significantly for congruent versus incongruent trials for M11 but not for M12, as shown in Fig 4 (t-test 200ms before bar release, *p* = 1.723 × 10^−49^ for M11, *p* = 0.0993 for M12).

This neural correlate of response-hemifield congruence is interesting given LIP’s role in attention. Below, we will show that a similar effect appears in the model due to interaction between attention and strengths of intrinsic connections among processing areas (potentially reflective of learning effects), and that this may explain M11’s significantly faster responses to congruent than incongruent trials as compared to M12 (Table 1). For more discussion of potential implications of this effect, see [14], where limb preferences are also described and both effects are studied in depth. Cells with limb preferences show significantly greater activity when the L or R limb responds (see S1 Text), but as described there we did not explicitly use this classification in our analyses or model.

#### Behavioral data show unusual accuracy versus reaction time effects

In fixed difficulty choice tasks, accuracy often increases as RTs increase, reflecting a speed-accuracy tradeoff [8, 17]. To examine this in the present task, we partitioned RTs into 100 ms bins for each set size and computed accuracies in each bin for response-hemifield congruent and incongruent trials: see Fig 5. M11 showed large *decreases* in accuracy with RT for congruent trials and a more complex rise and fall for incongruent trials, associated with a substantial set size effect and overall accuracy deficit in that condition (Table 1). M12 also showed decreases in accuracy, stronger for incongruent than congruent trials, but smaller overall than in M11, and with small set size effects on both congruent and incongruent trials.

This “reverse speed-accuracy tradeoff” is most evident in both monkeys for set sizes 4 and 6, for which RT distributions are broader. The set size effect is expected: when targets must be found among several distractors, search time increases and accuracy is likely to suffer. The overall decrease in accuracy with RT may reflect differences in experienced difficulty (e.g., due to lapses of attention), with more difficult trials showing longer RTs and lower accuracy.

### A multi-area, multi-stage accumulator model

As noted in the Introduction, previous binary choice models have employed pairs of leaky competing accumulators (see [7, 18] for reviews) or scalar drift-diffusion and Ornstein-Uhlenbeck processes [8, 9, 19, 20]. Such models can fit behavioral data and neural recordings during evidence integration (e.g., [6, 21]), but it seems unlikely that two accumulators, much less one representing the difference between activations in two populations, could reproduce the rich variety of LIP responses described above. Moreover, such models have been used to fit neural data from perceptual choice tasks in which a single stimulus was shown on each trial and responses were reported via saccades, in contrast to stimulus arrays in which the target must be located covertly and its identity signalled by lever release, as in the current task. Indeed, the FR patterns of Figs 2–4 show initial rises but then *decreases* in the ≈ 300 ms interval prior to response, compared with the monotonic rise to threshold typical of LIP cells before saccades in simpler choice tasks [22]. Furthermore, the normal speed-accuracy tradeoff is reversed.

#### Components of the model

**Fig 6:**
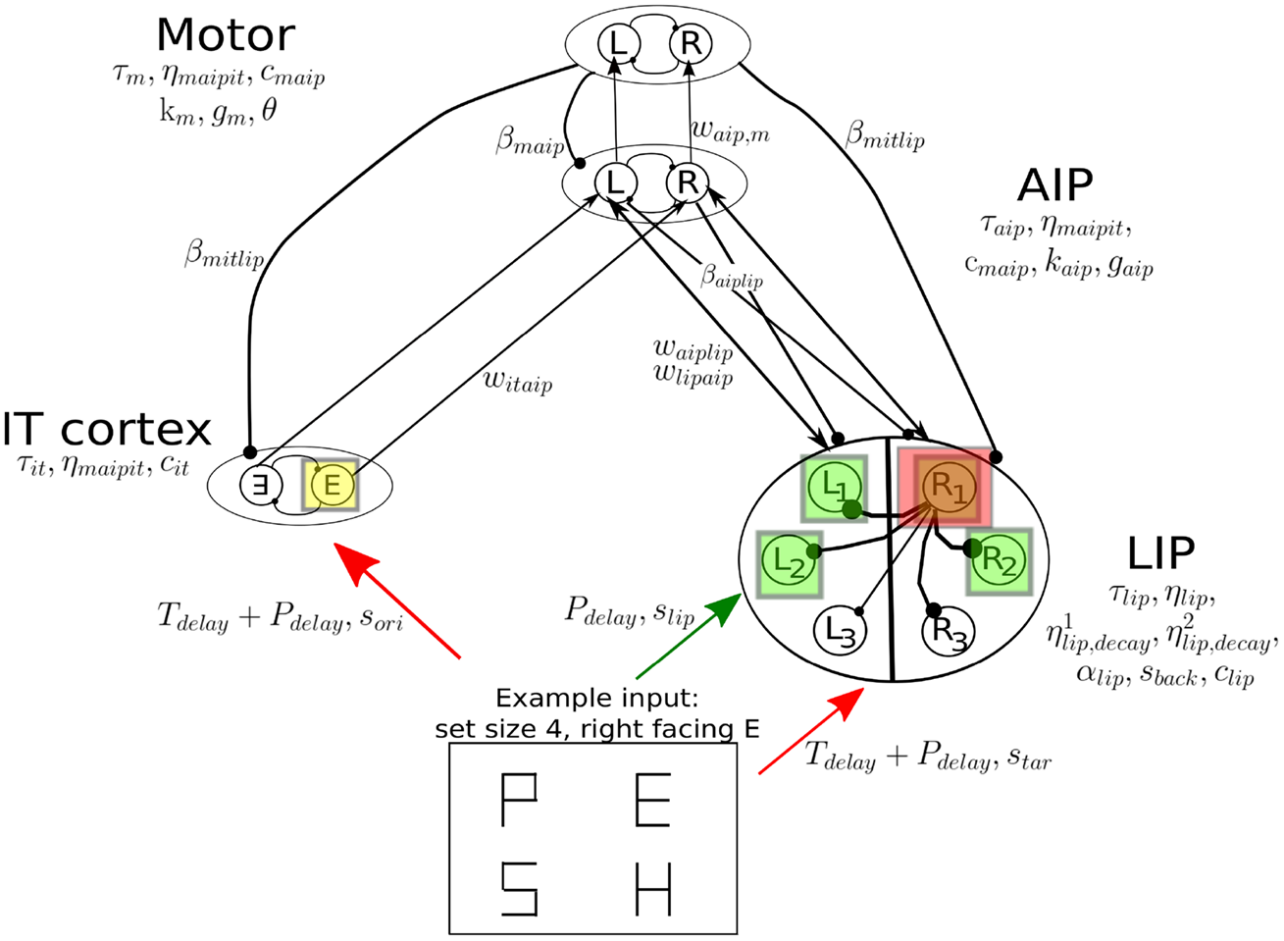
The network model for the covert search task. There are four main components: LIP, a feature selective area (IT), a limb pre-motor area (AIP) and motor cortex. Boxes to the right of AIP and motor areas indicate that the units in these regions are gated accumulators. The box representing stimulus display illustrates how, following cue onset at *t* = 0, inputs enter IT and LIP for set size 4 and target a right-facing 𝖤 in the upper right LIP unit’s receptive field. Arrows and filled circles respectively indicate excitatory and inhibitory connections. Connection strengths *w*, *β*, time constants *τ*, leakages *k*, noise levels *c* and all other parameters are described in the text, along with further details and the roles of perceptual and target delays *P*_*delay*_ and *T*_*delay*_.

The considerations above, and the rationale for its structure and components provided under “Model construction” in the Results section below, led to the model shown in Fig 6. The areas labeled inferior temporal cortex (IT) and anterior intraparietal area (AIP) respectively exemplify a feature-selective area in visual cortex and a limb pre-motor area. We use these labels for brevity, noting that other areas in visual cortex and elsewhere may be involved in place of IT and AIP.

The LIP area contains six units representing pools of neurons with RFs corresponding to stimulus locations. These pools mutually inhibit one another with connection strengths decaying as RFs grow farther apart as proposed in [12], where analogous data from frontal eye fields were successfully fitted. Net inhibition increases as more units activate, causing overall suppression of FRs and producing the set size effect.

Noisy stimulus inputs enter LIP successively in two stages, following an initial sensory latency *P*_*delay*_ due to signal transmission from the retina to parietal lobe. First an input of mean strength *s*_*lip*_ is fed to 2, 4 or 6 LIP units, depending upon set size. This represents a general perceptual signal driven by the placeholders prior to cue onset. After a further delay *T*_*delay*_ to account for search time to find the target, which we suppose is achieved in another (possibly frontal) brain area, a second input of mean strength *s*_*tar*_ is added to the unit whose RF corresponds to target location. Simultaneous with this input to LIP at *t* = *P*_*delay*_ + *T*_*delay*_, a separate input of mean strength *s*_*ori*_ enters one of the two mutually inhibiting units representing target orientation in IT cortex; in congruent trials *s*_*ori*_ enters on the same side as the LIP input *s*_*tar*_, and in incongruent trials on the opposite side. No additional delay parameter for the orientation selection process prior to or in IT is included because we assume that this process occurs quickly relative to the target identification process in LIP. In preliminary work [23] we analyzed the set size 4 RT data in a manner similar to that of [24]. We found no evidence of systematic search strategies and so do not explicitly model the target search process.

All input signals are piecewise-constant step functions filtered through Ornstein-Uhlenbeck (OU) processes to represent stimuli passed through synapses, as used previously in reductions of spiking neuron models of perceptual decisions [25, 26]. The filtered signals are combined with mutual inhibition and feedback and passed to their respective LIP and IT units through a firing rate input-output function derived in [26]. Such functions have been shown to be equivalent to other popular activation level models [27].

Area IT feeds forward via excitatory connections to a pair of gated accumulator units representing AIP, and LIP projects to AIP through mutually excitatory connections according to which hemifield the LIP unit belongs. Area AIP also projects back to LIP via cross inhibition, with the left AIP unit inhibiting all right LIP units, and vice-versa. Via this pathway LIP also reflects the activity of AIP. The AIP units are similar to the gated accumulators of [12]; in that paper signals derived from FEF recordings were passed through such units in order to fit RTs. Gated accumulators also play the role of bistable neurons in [28].

The AIP units project forward via excitatory connections to a second pair of mutually inhibitory accumulator units, equipped with response thresholds. As activity grows in this motor layer, all other areas receive inhibition that strengthens substantially as one unit’s FR approaches and crosses threshold. This forms a reset mechanism that drives down IT and LIP FRs, as suggested in previous decision making models [28]. Attention suppression has also been observed in EEG event-related potentials, and has been proposed as a general-purpose mechanism to terminate the allocation of attention [29]. When the first motor unit reaches threshold, the corresponding response is made and the RT is logged, after incorporating a nondecision time *T*_0_ representing motor latency.

### The model equations

We now present the stochastic differential equations (SDEs) that define the model in explicit detail. The parameters to be fitted are listed in Table 2 and explained below. All other parameters are fixed at constant values, also given below. Throughout *ξ*(*t*) denotes an *independent* (i.i.d.) realization of white noise with mean zero and unit variance in each equation. We use (⋅) to denote normal parentheses and [⋅] to denote arguments in functions; *H*[⋅] denotes the Heaviside function

$$\begin{array}{c} H\left[x\right]=\{\begin{array}{cc} 1 & :x\geq 0,\\ 0 & :x<0. \end{array} \end{array}$$ (1)

**Table 2:** List of model parameters.

**P**_**delay**_	**perceptual delay**
**T**_**delay**_	**target delay**
*τ*_*m*_	motor units time constant
*τ*_*it*_	IT units time constant
*τ*_*aip*_	AIP units time constant
*τ*_*lip*_	LIP units time constant
*η*_*maipit*_	mutual inhibition within motor, AIP, and IT
***η***_**lip**_	**inhibition strength in LIP**
$\eta_{lip,decay}^{1}$	spatial decay of inhibition for units one-step removed in LIP
$\eta_{lip,decay}^{2}$	spatial decay of inhibition for units two-steps removed in LIP
*α*_*lip*_	recurrent excitation in LIP
*w*_*aipm*_	excitatory connection weight for AIP → motor
**w**_**itaip**_	**excitatory connection weight for IT → AIP**
**w**_**lipaip**_	**excitatory connection weight for LIP → AIP**
**w**_**aiplip**_	**excitatory connection weight for AIP → LIP**
*β*_**aiplip**_	**inhibitory crossed connection weight for AIP → LIP**
*β*_*maip*_	inhibitory weight for motor → AIP
*β*_*mitlip*_	inhibitory weight for motor → IT and LIP
**s**_**lip**_	**strength of perceptual input to all LIP units**
**s**_**tar**_	**strength of target input to target LIP unit**
*s*_*back*_	strength of background noise inputs to LIP
*s*_*ori*_	strength of target orientation input to IT
*c*_*maip*_	noise standard deviation for motor, AIP
**c**_**it**_	**noise standard deviation for IT**
**c**_**lip**_	**noise standard deviation for LIP**
*k*_*m*_	motor leak parameter
*g*_*m*_	motor gate parameter
*k*_*aip*_	AIP leak parameter
*g*_*aip*_	AIP gate parameter
**T**_**0**_	**nondecision time (primarily motor latency)**
*θ*	**motor layer threshold**

31 model parameters must be fit to the data for each monkey. 18 of these were fit simultaneously to the data for both monkeys (normal font), while 13 were allowed to vary between M11 and M12 (bold font), resulting in a total of 44 free parameters fit over all experimental conditions.

Some notation is necessary to define how equations depend upon the trial inputs. We denote the input array by a triplet 𝓐 = (set size, position, 𝖤-orientation) = (𝓐_*ss*_, 𝓐_*p*_, 𝓐_𝖤_), indicating set size, position and 𝖤-orientation of the target. set size 𝓐_*ss*_ is either 2, 4, or 6. The position 𝓐_*p*_ ∈ {*L*1, *L*2, *L*3, *R*1, *R*2, *R*3} indicates whether the target is on the left or right, and in the top, middle or bottom rows as per the labels on the LIP units of Fig 6. The orientation 𝓐_𝖤_ indicates if the target is left- (∃) or right-facing (𝖤). E.g., if set size is 4 and the target is a right-facing 𝖤 at upper right, 𝓐 = (4, *R*1, 𝖤). If set size is 6 and the target is a left-facing 𝖤 in the middle row, left side, 𝓐 = (6, *L*2, ∃).

The model consists of 20 SDEs. Activities of the six LIP units are denoted by *X*_*lip*, *L*1_, *X*_*lip*, *L*2_, *X*_*lip*, *L*3_, *X*_*lip*, *R*1_, *X*_*lip*, *R*2_, and *X*_*lip*, *R*3_. Subscripts are chosen to indicate the location of each LIP unit’s receptive field in the same manner as for 𝓐, i.e. *X*_*lip*, *R*2_ is the state variable for the LIP unit with a receptive field in the middle row on the right side of the hemifield. Each LIP unit has an input OU process paired with it, denoted by *ρ*_*lip*, *L*1_, *ρ*_*lip*, *L*2_, *ρ*_*lip*, *L*3_, *ρ*_*lip*, *R*1_, *ρ*_*lip*, *R*2_, and *ρ*_*lip*, *R*3_, with subscripts indicating the pairings. There are two IT units, one for each possible 𝖤-orientation denoted by *X*_*it*, 𝖤_ for the right facing 𝖤 and *X*_*it*, ∃_ for the left facing ∃; each IT unit also has an input OU process, denoted by *ρ*_*it*, 𝖤_ and *ρ*_*it*, ∃_. AIP contains two units, one for each possible response, denoted by *X*_*aip*, *L*_ for left and *X*_*aip*, *R*_ for right responses. The motor area also contains two units, *X*_*m*, *L*_ for left and *X*_*m*, *R*_ for the right response.

The LIP and IT units use the input-output function *ϕ*[*I*] derived in [26] Eq (24)] with the parameter values specified in [26] Appendix A]:

$$\begin{array}{c} \phi \left[I\right]=0.001+\frac{0.352(I-0.384)}{1-\text{exp}[-352(I-0.384)]+0.352(I-0.384)/0.1}. \end{array}$$ (2)

We now state the equations for the six LIP units:

$$\begin{array}{ccc} \tau_{lip}\frac{dX_{lip,L1}}{dt} & = & \phi [-\beta_{aiplip}X_{AIP,R}+w_{aiplip}X_{AIP,L}+\alpha_{lip}X_{lip,L1}-I_{mi}-\eta_{lip}\left(X_{lip,R1}+X_{lip,L2}\right)\\ & & -\eta_{lip,decay}^{1}\times \eta_{lip}\left(X_{lip,R2}+X_{lip,L3}\right)-\eta_{lip,decay}^{1}\times \eta_{lip,decay}^{2}\times \eta_{lip}X_{lip,R3}+\rho_{lip,L1}]\\ \tau_{lip}\frac{dX_{lip,L2}}{dt} & = & \phi [-\beta_{aiplip}X_{AIP,R}+w_{aiplip}X_{AIP,L}+\alpha_{lip}X_{lip,L2}-I_{mi}-\eta_{lip}\left(X_{lip,L1}+X_{lip,L3}\right)\\ & & -\eta_{lip,decay}^{1}\times \eta_{lip}\left(X_{lip,R1}+X_{lip,R3}\right)-\eta_{lip,decay}^{1}\times \eta_{lip,decay}^{2}\times \eta_{lip}X_{lip,R2}+\rho_{lip,L2}]\\ \tau_{lip}\frac{dX_{lip,L3}}{dt} & = & \phi [-\beta_{aiplip}X_{AIP,R}+w_{aiplip}X_{AIP,L}+\alpha_{lip}X_{lip,L3}-I_{mi}-\eta_{lip}\left(X_{lip,L2}+X_{lip,R3}\right)\\ & & -\eta_{lip,decay}^{1}\times \eta_{lip}\left(X_{lip,L1}+X_{lip,R2}\right)-\eta_{lip,decay}^{1}\times \eta_{lip,decay}^{2}\times \eta_{lip}X_{lip,R1}+\rho_{lip,L3}]\\ \tau_{lip}\frac{dX_{lip,R1}}{dt} & = & \phi [-\beta_{aiplip}X_{AIP,L}+w_{aiplip}X_{AIP,R}+\alpha_{lip}X_{lip,R1}-I_{mi}-\eta_{lip}\left(X_{lip,L1}+X_{lip,R2}\right)\\ & & -\eta_{lip,decay}^{1}\times \eta_{lip}\left(X_{lip,L2}+X_{lip,R3}\right)-\eta_{lip,decay}^{1}\times \eta_{lip,decay}^{2}\times \eta_{lip}X_{lip,L3}+\rho_{lip,R1}]\\ \tau_{lip}\frac{dX_{lip,R2}}{dt} & = & \phi [-\beta_{aiplip}X_{AIP,L}+w_{aiplip}X_{AIP,R}+\alpha_{lip}X_{lip,R2}-I_{mi}-\eta_{lip}\left(X_{lip,R1}+X_{lip,R3}\right)\\ & & -\eta_{lip,decay}^{1}\times \eta_{lip}\left(X_{lip,L1}+X_{lip,L3}\right)-\eta_{lip,decay}^{1}\times \eta_{lip,decay}^{2}\times \eta_{lip}X_{lip,L2}+\rho_{lip,R2}]\\ \tau_{lip}\frac{dX_{lip,R3}}{dt} & = & \phi [-\beta_{aiplip}X_{AIP,L}+w_{aiplip}X_{AIP,R}+\alpha_{lip}X_{lip,R3}-I_{mi}-\eta_{lip}\left(X_{lip,L3}+X_{lip,R2}\right)\\ & & -\eta_{lip,decay}^{1}\times \eta_{lip}\left(X_{lip,L2}+X_{lip,R1}\right)-\eta_{lip,decay}^{1}\times \eta_{lip,decay}^{2}\times \eta_{lip}X_{lip,L1}+\rho_{lip,R3}] \end{array}$$ (3)

where *I*_*mi*_, defined as

$$\begin{array}{c} I_{mi}=\beta_{mitlip}(X_{m,L}+X_{m,R})), \end{array}$$ (4)

is the global motor inhibition signal common to all LIP units.

Next we state the six OU processes which provide stimulus input to each LIP unit:

$$\begin{array}{ccc} \tau_{lip,noise}\frac{d\rho_{lip,LOC}}{dt} & = & -\rho_{lip,LOC}+I_{per}\left[{𝓐}_{ss},LOC\right]H\left[t-P_{delay}\right]\\ & & +I_{tar}\left[{𝓐}_{p},LOC\right]H\left[t-P_{delay}-T_{delay}\right]+s_{back}+c_{lip}\;\xi_{lip,LOC}\left(t\right), \end{array}$$ (5)

where *LOC* ∈ {*L*1, *L*2, *L*3, *R*1, *R*2, *R*3} denotes one of the locations for the LIP units, and *ξ*_*lip*, *LOC*_(*t*) denotes independent additive white noise with zero mean and unit variance input to each unit. Guided by synaptic time constants for fast neurotransmitters (*AMPA*, *GABA*_*A*_), we fix the time constant for these OU processes at *τ*_*lip*, *noise*_ = 5 ms. This is faster than the 2 ms time constants used in [25, 26] but necessary to allow the relatively coarse temporal resolution *dt* = 0.5 ms used in simulations (see below section on fitting methods). The perceptual input from the stimulus *I*_*per*_[𝓐_*ss*_, *LOC*] activates the appropriate number of OU processes depending upon set size:

$$\begin{array}{c} I_{per}\left[{𝓐}_{ss},LOC\right]=\{\begin{array}{cc} s_{lip} & \;\text{if}\;{𝓐}_{ss}=2\;\text{and}\;LOC\in \left\{L1,R1\right\},\\ s_{lip} & \;\text{if}\;{𝓐}_{ss}=4\;\text{and}\;LOC\in \left\{L1,R1,L2,R2\right\},\\ s_{lip} & \;\text{if}\;{𝓐}_{ss}=6,\\ 0 & \;\text{otherwise}\;. \end{array} \end{array}$$

This states that the perceptual signal is sent units L1 and R1 for set size 2, L1, R1, L2 and R2 for set size 4, and all 6 for set size 6. All LIP units are active for all set sizes, including those that do not receive perceptual input. The target input *I*_*tar*_[𝓐_*p*_, *LOC*] activates only the unit which corresponds to the target location:

$$\begin{array}{c} I_{tar}\left[{𝓐}_{p},LOC\right]=\{\begin{array}{cc} s_{tar} & \;\text{if}\;{𝓐}_{p}=LOC,\\ 0 & \;\text{otherwise}\;. \end{array} \end{array}$$

We now state the equations for IT:

$$\begin{array}{ccc} \tau_{it}\frac{dX_{it,\exists }}{dt} & = & \phi [\rho_{it,\exists }-\eta_{maipit}X_{it,𝖤}-I_{mi}]-X_{it,\exists },\\ \tau_{it}\frac{dX_{it,𝖤}}{dt} & = & \phi [\rho_{it,𝖤}-\eta_{maipit}X_{it,\exists }-I_{mi}]-X_{it,𝖤}, \end{array}$$ (6)

where *I*_*mi*_ is defined in Eq 4. The equations for the two input OU processes to the IT units are

$$\begin{array}{ccc} \tau_{it,noise}\frac{d\rho_{it,ORI}}{dt} & = & -\rho_{it,ORI}+I_{ori}\left[{𝓐}_{𝖤},ORI\right]H\left[t-P_{delay}-T_{delay}\right]+c_{it}\;\xi_{it,ORI}\left(t\right). \end{array}$$ (7)

Here *ORI* ∈ {𝖤, ∃} denotes one of the two possible E orientations and we fix the time constant of this OU process at *τ*_*it*, *noise*_ = 5 ms, as for *τ*_*lip*, *noise*_ above. As above *ξ*_*it*, *ORI*_(*t*) is additive white noise with zero mean and unit variance. The target input from the stimulus *I*_*ori*_[𝓐_𝖤_, *ORI*] is

$$\begin{array}{c} I_{ori}\left[{𝓐}_{𝖤},ORI\right]=\{\begin{array}{cc} s_{ori} & \;\text{if}\;{𝓐}_{𝖤}=ORI,\\ 0 & \;\text{otherwise}\;. \end{array} \end{array}$$

Before writing the equations for AIP and motor units, we specify the piecewise-linear gating function *G*[⋅, ⋅] used in both these areas:

$$\begin{array}{c} G\left[I,g\right]=\{\begin{array}{cc} I-g & \;\text{if}\;I-g>0,\\ 0 & \;\text{otherwise}. \end{array} \end{array}$$ (8)

The equations for AIP may now be written:

$$\begin{array}{ccc} \tau_{aip}\frac{dX_{aip,L}}{dt} & = & -k_{aip}X_{aip,L}-\eta_{maipit}X_{aip,R}+G[w_{itaip}X_{it,\exists }\\ & & +w_{lipaip}\left(X_{lip,L1}+X_{lip,L2}+X_{lip,L3}\right)-I_{maip},g_{aip}]+c_{maip}\;\xi_{aip,L}\left(t\right),\\ \tau_{aip}\frac{dX_{aip,R}}{dt} & = & -k_{aip}X_{aip,R}-\eta_{maipit}X_{aip,L}+G[w_{itaip}X_{it,E}\\ & & +w_{lipaip}\left(X_{lip,R1}+X_{lip,R2}+X_{lip,R3}\right)-I_{maip},g_{aip}]+c_{maip}\;\xi_{aip,R}\left(t\right), \end{array}$$ (9)

where *I*_*maip*_ is the same as in Eq 4 but with *β*_*mitlip*_ replaced with *β*_*maip*_. Finally, the equations for the motor units are

$$\begin{array}{ccc} \tau_{m}\frac{dX_{m,L}}{dt} & = & -k_{m}X_{m,L}-\eta_{maipit}X_{m,R}+G\left[w_{aipm}X_{aip,L},g_{m}\right]+c_{maip}\;\xi_{m,L}\left(t\right),\\ \tau_{m}\frac{dX_{m,R}}{dt} & = & -k_{m}X_{m,R}-\eta_{maipit}X_{m,L}+G\left[w_{aipm}X_{aip,R},g_{m}\right]+c_{maip}\;\xi_{m,R}\left(t\right). \end{array}$$ (10)

Again the terms *ξ*_*aip*, *R*_(*t*) and *ξ*_*m*, *L*, *R*_(*t*) denote independent white noise processes with zero mean and unit variance. Motor unit FRs were capped at 150 Hz by setting *X*_*m*, *L*_ and *X*_*m*, *R*_ at that value whenever they exceeded it; in practice these FRs rarely exceeded 120 Hz.

In total the system is defined by 20 SDEs: 6 LIP units, 6 LIP OU processes, 2 IT units, 2 IT OU processes, 2 AIP units, and 2 motor units.

### Model fitting methods

To fit model parameters, we numerically optimized an objective function that accounts for errors in both FRs and behavioral data:

$$\begin{array}{ccc} & & \alpha_{FR}\frac{1}{2\cdot SS}\sum_{cong,incong}\sum_{j=1}^{SS}\sum_{t=0}^{800}{\left(\frac{FR_{t}^{j}-{\bar{FR}}_{t}^{j}}{FR_{t}^{j}}\right)}^{2}\\ & & \;+\alpha_{corr}\frac{1}{2}\sum_{cong,incong}\chi_{correct}^{2}\\ & & \;+\alpha_{incorr}\frac{1}{2}\sum_{cong,incong}\chi_{incorrect}^{2}\;. \end{array}$$ (11)

The first term in Eq (11) represents the error between $FR_{t}^{j}$ simulated in the model and those in the data ${\bar{FR}}_{t}^{j}$ for each LIP unit (indexed by RF *j*), summed over the time interval [0, 800] ms from cue onset, and over the relevant RFs for the set size (*SS*). Seeking optima for all trial conditions, we summed errors between model results and data in response-hemifield congruent and incongruent cases, with the model’s LIP and IT areas receiving inputs as described in the Results section. Data for each LIP unit fit was computed by averaging FRs over the appropriate trials in which the recorded cell represented the corresponding LIP model unit (e.g., data from a trial simulated with left-facing ∃ in unit L1 was matched to averaged FRs of trials in which the target was in that RF; cf. Fig 6). Other model LIP units were matched to corresponding FRs of trials with distractors in their RFs.

The second and third terms describe the *χ* ^2^ error for RTs on correct and incorrect trials, computed using the chi-square fitting method of [30] implemented in MATLAB using codes custom written by the authors (MS and SF). This uses the 0.1, 0.3, 0.5, 0.7, and 0.9 quantiles from the observed RT distribution to define six bins, of which the fastest and slowest each contain 10% of the total number of trials, and the central 4 each contain 20%. RTs predicted by model simulations were then collected in the same bins, and each *χ* ^2^ is computed as:

$$\begin{array}{c} \chi^{2}=\sum_{bin=1}^{6}\frac{{\left(\text{data}\;{\text{trials}}_{bin}-\text{model}\;{\text{trials}}_{bin}\right)}^{2}}{\text{model}\;{\text{trials}}_{bin}}. \end{array}$$ (12)

Both congruent and incongruent trials were included in Eq (12), as described above for FRs, but separate histograms were fitted for correct and error RTs to account for accuracy.

The different physical units of neural and behavioral data are accommodated in Eq (11) by nondimensionalization, and weight coefficients *α*_*FR*_, *α*_*corr*_, and *α*_*incorr*_ are used to obtain comparable fit qualities for both data sets. Specifically, the values *α*_*corr*_ = *α*_*incorr*_ = 1, and *α*_*FR*_ = 0.25 were found to give all terms in Eq (11) similar orders of magnitude at starting points for the optimization algorithm. These values were used to produce all fitting results.

For each call to the objective function, we numerically simulated 6000 trials of the model using a stochastic Runge-Kutta algorithm implemented in custom software written in C and MATLAB by the authors (MS and SF). Temporal resolution was *dt* = 0.5 ms in all simulations. From these simulations we computed averaged FRs for each LIP unit and RT quantiles for correct and incorrect trials to generate the *χ* ^2^ error of Eq (11). Good starting points were found by hand, mainly by adjusting parameters until model results were reasonably close to the data (poor starting points caused the optimization to terminate immediately). Given such points, the model was simultaneously fitted to data from M11 and M12 using a global optimization algorithm. As noted in Table 2, we constrained 18 of the 31 free model parameters to be equal for M11 and M12 and allowed 13—those we think most likely responsible for differences in the animals’ behaviors—to vary between them, for a total of 44 to be fit for the two monkeys.

We tried several optimization algorithms with limited success, including MATLAB’s fminsearch, fmincon, PSWARM and simulated annealing. The parallelized routine HOPSPACK [31] improved fine-tuning of parameter sets, although search resolution was coarse and convergence not as tight as we wished. A combination of simulated annealing and the bounded derivative-free optimization routine fminsearchbnd, both implemented in Matlab and available from the Matlab file exchange [32, 33], gave our best results as reported in the main text. We suspect that stochasticity in both model and data makes our objective function bumpy, resulting in poor convergence of the optimization routines. Use of efficiently-parallelized codes and many processors did not alleviate these difficulties.

## Results

Before presenting and interpreting fits of the model to the data set described in “Analyses and Methods,” we outline our rationale for selecting the model’s architecture and components, since these are also results of our study.

### Model construction

In constructing the model of Fig 6 we sought the simplest architecture consistent with the major findings of [14, 15], and in particular differences between the electrophysiological and behavioral data for M11 and M12 as described in “The covert search task” above. We summarize the main points here; model details appear under “A multi-area, multi-stage, accumulator model.”

A correct choice requires the monkeys to select the target, determine its orientation and release the appropriate paw. Although the FR data of Fig 4 suggests that LIP neurons encode correlates of all these processes, evidence from reversible inactivation experiments suggests that its primary role was in target selection [14]. In [34] LIP was inactivated in one hemisphere by muscimol injection. This impaired the monkeys’ ability to locate the target in the contralateral hemifield, but not their ability to respond with a specific paw, suggesting that LIP activity associated with the manual release may not play a central role in decisions, but reflect computations that are at least partially performed in other areas depending on the requirements of the task. Indeed, there is increasing evidence that other areas, including superior colliculus, prefrontal cortex, frontal eye fields and caudate, are involved in perceptual decisions, e.g. [1, 35–37].

We therefore assume that LIP encodes target/distractor identities, but that orientation information is accumulated in a feature-selective area in visual cortex, and that both LIP and this visual area feed forward to a limb pre-motor area that in turn connects with motor cortex to generate the manual response. We make no specific claims about the identity of the visual and premotor areas, but model the former based on published reports about inferior temporal (IT) cortex or area V4, which are known to encode complex shapes (see e.g. [38–42] and [43, 44]), and the latter on the anterior intraparietal area (AIP), which is anatomically adjacent to LIP and contains visual and grasp-responsive cells [45, 46]. We also assume that the orientation selection process in the feature-selective area (IT) occurs rapidly relative to target identification in LIP because orientation detection simply requires a choice between two non-noisy options.

In order to produce the target/distractor and congruence effect with target in LIP RF for M11 (Fig 4), we include recurrent excitatory and cross-inhibitory connections in the LIP pathway, but we keep the feature-selective pathway and premotor-motor connections purely feedforward and enforce bilateral (L/R) symmetry (Fig 6). We also exclude direct connections between LIP and the feature-selective area. Even with this (relatively) minimalist architecture the model has 31 free parameters, as noted in Table 2.

### Fits to set size 4 data

We first describe the results of model fits to the set size 4 data for M11 and M12, and then discuss predictions made by these two models, with all parameters fixed, for set sizes 4 and 6.

We fit the model simultaneously to the set size 4 data for both monkeys, allowing 13 of the 31 parameters to vary across M11 and M12 as noted in Table 2. The model was run under both congruent and incongruent response-hemifield conditions and fitting errors computed from Eq (11). To simulate these conditions, we respectively input a rightwards facing 𝖤 to the upper right or left LIP unit in Fig 6. This configuration sufficed because data FRs were averaged over multiple RF configurations and model parameters are the same across both hemispheres. Different model LIP unit average FRs were then compared to corresponding FRs from data: e.g., in the congruent condition, the upper right LIP unit was compared with target in RF FRs, upper left and middle LIP units were compared with distractor in RF in hemifield opposite to target FRs, and the middle right LIP unit was compared with distractor in RF in same hemifield as target FRs. The lower two LIP units had no stimulus input and while they remain in the model for all set sizes, their FRs were excluded from the comparisons.

Table 3 shows the resulting parameter values for each monkey. Model accuracies and mean RTs are given in Table 4 in the same format as Table 1, along with the set size 2 and 6 predictions discussed below. Tables 5 and 6 compare model accuracy and mean RTs to each monkey’s data for all set sizes in congruent and incongruent conditions.

**Table 3:** Model parameter values fitted to the set size 4 data.

	M11	M12
**P**_**delay**_	**24.08**	**44.58**
**T**_**delay**_	**55.72**	**48.20**
*τ*_*m*_	438.2	
*τ*_*it*_	68.66	
*τ*_*aip*_	119.7	
*τ*_*lip*_	34.80	
*η*_*maipit*_	5.371	
***η***_**lip**_	**2.936**	**0.1032**
$\eta_{lip,decay}^{1}$	0.8470	
$\eta_{lip,decay}^{2}$	0.1300	
*α*_*lip*_	1.469	
*w*_*aipm*_	4.827	
**w**_**itaip**_	**10.98**	**18.75**
**w**_**lipaip**_	**6.495**	**2.520**
**w**_**aiplip**_	**6.276**	**1.166**
*β*_**aiplip**_	**8.422**	**4.266**
*β*_*maip*_	3.108	
*β*_*mitlip*_	11.02	
**s**_**lip**_	**0.6317**	**0.4088**
*s*_*tar*_	0.3997	0.4165
*s*_*back*_	0.5038	
*s*_*ori*_	1.227	
*c*_*maip*_	0.7866	
**c**_**it**_	**1.465**	**0.6744**
*c*_*lip*_	1.950	1.818
*k*_*m*_	0.0000	
*g*_*m*_	0.1079	
*k*_*aip*_	12.51	
*g*_*aip*_	0.0005	
**T**_**0**_	**169.8**	**96.17**
*θ*	0.0926	0.0961

Parameter fits to set size 4 data. In addition to the 18 parameters constrained to be equal for M11 and M12, three more differ by under 7.3% (*s*_*tar*_, *c*_*lip*_, *θ*, normal font). Timescales are in msec, and the motor threshold *θ* in kHz. The 10 parameters that significantly differentiate the monkeys (bold font) are discussed in the text.

**Table 4:** Model accuracy and mean reaction times.

	Monkey 11		Monkey 12	
	Accuracy	Mean RT	Accuracy	Mean RT
**Set size 2**	96.7%	560.7 ± 2.4	98.8%	496.5 ± 2.5
**Set size 4**	94.1%	491.9 ± 2.1	98.7%	470.1 ± 2.4
**Set size 6**	87.1%	453.2 ± 2.1	98.9%	447.1 ± 2.2
**Congruent**	95.6%	476.3 ± 1.6	98.8%	460.8 ± 1.9
**Incongruent**	89.4%	526.7 ± 2.1	98.7%	481.1 ± 2.0

Accuracy and mean reaction times (RTs) for different set sizes from the model for each monkey. Congruent trials were computed by averaging over all set sizes, as in Table 1. RTs are given in ms ± SEM.

**Table 5:** Comparison of monkey 11 accuracy and mean reaction time data and model predictions.

	Congruent		Incongruent	
	Accuracy	Mean RT	Accuracy	Mean RT
**Set size 2**	97.4 / 97.9%	437.6±4.2 / 525.0±2.8	93.5 / 95.3%	500.2±5.7 / 599.2±3.6
**Set size 4**	95.8 / 96.7%	444.3±4.0 / 464.5±2.4	81.5 / 91.4%	537.2±5.1 / 520.0±3.2
**Set size 6**	92.4 / 92.2%	457.6±3.4 / 440.2±2.6	73.5 / 82.0%	549.0±4.6 / 466.4±3.1

Monkey 11 accuracy and mean reaction time (RT) data / model output for different set sizes, each separated into response-hemifield congruent versus incongruent conditions. RTs are given in ms ± SEM.

**Table 6:** Comparison of monkey 12 accuracy and mean reaction time data and model predictions.

	Congruent		Incongruent	
	Accuracy	Mean RT	Accuracy	Mean RT
**Set size 2**	98.0 / 98.72%	468.7±8.1 / 485.5±3.4	98.2 / 98.8%	455.1±7.4 / 507.8±3.7
**Set size 4**	96.2 / 98.7%	491.8±6.8 / 461.4±3.3	95.9 / 98.6%	488.0±6.0 / 478.9±3.4
**Set size 6**	94.1 / 99.1%	506.7±7.5 / 436.6±3.0	93.1 / 98.8%	499.7±6.4 / 457.8±3.2

Monkey 12 accuracy and mean reaction time (RT) data / model output for different set sizes, each separated into response-hemifield congruent versus incongruent conditions. RTs are given in ms ± SEM.

**Fig 7:**
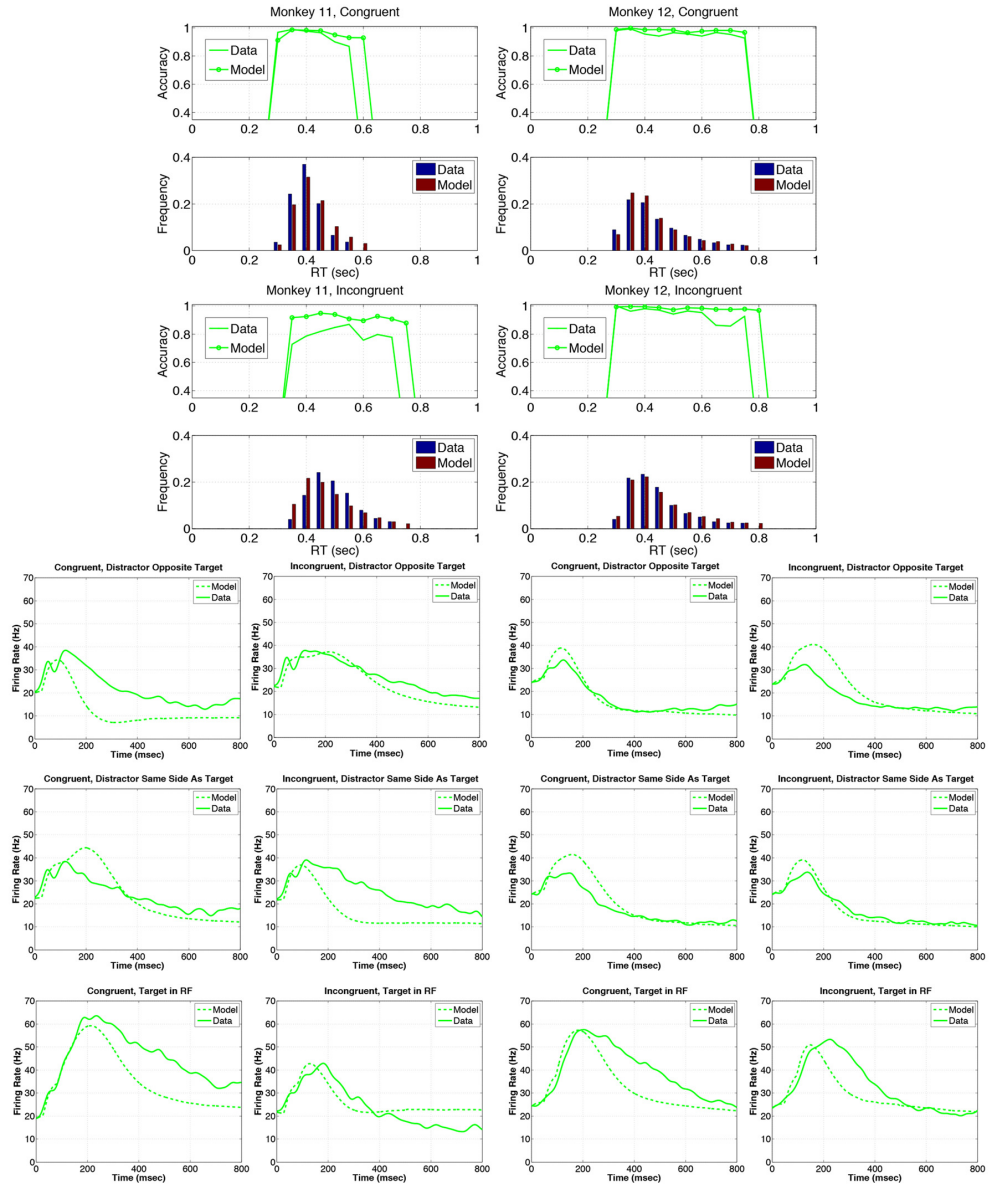
Model fits to the set size 4 data. Model parameters are given in Table 3. M11 data and model fits are given in the left panels in rows 1–4, and in the first and second columns of rows 5–7. Similarly, M12 data and model fits are given in the right panels of rows 1–4, and in the third and fourth columns of rows 5–7. Rows 1–2 show accuracy and reaction time (RT) histograms with response-hemifield congruent inputs, while rows 3–4 show histograms for response-hemifield incongruent inputs. In accuracy versus RT plots, solid traces indicate data and circles denote model output; in RT histograms, blue bars indicate data and red bars show model output. Rows 5,6 and 7 respectively show firing rates (FRs) for response-hemifield congruent and incongruent inputs, with distractor in receptive field (RF) opposite to target hemifield, distractor in RF on same side as target, and target in RF. Model FRs are shown dashed and data as solid traces.

Fig 7 shows that the model captures the major features of the data. RT distributions and accuracy versus RT plots are well approximated for M12 in both conditions, excepting the last 4 bins in the incongruent condition (11.7% of responses), and M11’s accuracy is over predicted in the last 3 bins of the congruent condition (19.2% of responses). The model captures the downward trends in accuracy with increasing RT for both monkeys (Fig 7 rows 1 and 3), overestimating accuracy by 10% for M11’s incongruent trials, but only by 0.9—2.5% for the other 3 cases. Mean RTs are underestimated by 1.9—6.2% and overestimated by 4.5% for M11’s congruent trials (see set size 4 data in Table 5).

Fig 7 (rows 5–7) shows that LIP FRs rise higher with target in RF than with distractor in RF, and overall FRs are higher in M11 than M12 on all conditions, as in the data, except with distractor opposite target. However, the model’s FRs decay too rapidly for M11 on congruent trials with distractor in RF opposite target and target in RF, and for distractor in RF on the same side as target on incongruent trials. FR decays are also too rapid for M12 with target in RF on both congruent and incongruent trials. Although RT’s are reproduced well, the model systematically overestimates accuracy, while generally underestimating FRs. At the end of this section and in the Discussion we note some modifications and additions to the model that might remedy these discrepancies.

As described above, 18 parameters were held equal across both monkeys, including time constants, several excitatory and inhibitory weights, some input strengths, and internal parameters for the AIP and motor areas (unbolded entries in Table 3). Three parameters that were allowed to vary differ by under 7.3% (*s*_*tar*_, *c*_*lip*_ and *θ*, also unbolded). The remaining 10 parameters significantly differentiate M11 and M12 as we now describe, first considering connection weights, input strength *s*_*lip*_, and noise level *c*_*it*_.

**Fig 8:**
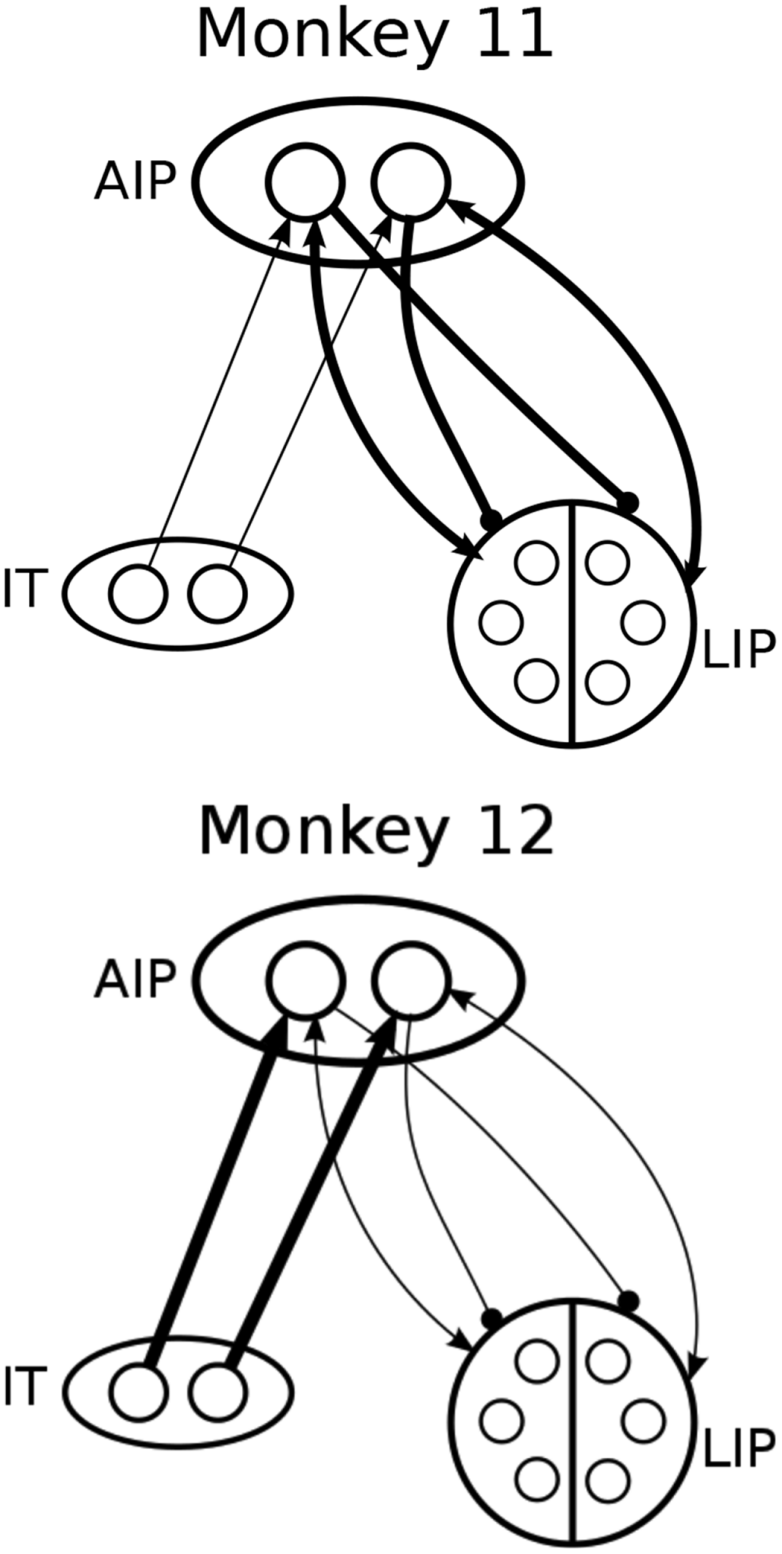
Network cartoon illustrating fitted connection weights among IT, AIP and LIP. Bold lines denote stronger fitted weights, predicting that monkey 11 has stronger connections between AIP and LIP, while monkey 12 has a stronger connections between IT and AIP.

Excitatory weights *w*_*lipaip*_ (6.495 > 2.520), *w*_*aiplip*_ (6.276 > 1.166) between LIP and AIP and cross-inhibition *β*_*aiplip*_ (8.422 > 4.266) from AIP to LIP are much stronger in M11 than M12, as is inhibition within LIP *η*_*lip*_ (2.936 > 0.1032; see boldface entries in Table 3 and network cartoons of Fig 8). The nonspecific LIP input *s*_*lip*_ is also stronger for M11 (0.6317 > 0.4088). In contrast, the excitatory weight from IT to AIP *w*_*itaip*_ is stronger in M12 than M11 (18.75 > 10.98). These differences produce higher LIP FRs for M11 than M12 in all six conditions, as shown in Fig 7 (rows 5 to 7).

These parameter values provide insights into mechanisms that cause differences in LIP FR patterns and their relationship to behavioral differences observed between the two animals. Specifically, the relative dominance of the LIP-AIP pathway in M11 and the IT-AIP pathway in M12 account for the response-hemifield congruence effect noted earlier. On incongruent trials both AIP units receive target activation, one from IT and the other from LIP, causing competition, slower RTs and lower accuracy. Moreover, M11’s strong AIP-LIP back-excitation amplifies the influence of target location, and the substantially greater IT noise *c*_*it*_ (1.465 > 0.6744) further degrades M11’s ability to decode orientation. In congruent trials, M11’s strong LIP-AIP re-entrant connections produce higher FRs in target RF of LIP; assisted by the IT-AIP pathway, these yield responses that are faster than M12’s and almost as accurate. In M12, the dominant IT-AIP pathway, moderate AIP-LIP cross-inhibition and weak AIP-LIP back-excitation favor target orientation for both congruent and incongruent trials, consistent with M12’s greater accuracy and uniformity over all conditions. See Tables 5 and 6 and compare Tables 1 and 4. Ratios of M11’s and M12’s connection weights *w*_*itaip*_, *w*_*lipaip*_, *w*_*aiplip*_ and *β*_*aiplip*_ and mutual inhibition *η*_*lip*_ within LIP are larger than those of *s*_*lip*_ and *c*_*it*_, suggesting that the former parameters are primarily responsible.

The delay parameters *P*_*delay*_, *T*_*delay*_ and the nondecision time *T*_0_ also differ significantly between M11 and M12. These account for sensory and motor latencies and target search times (excluded from the model), and they allow estimation of *decision times* associated with task-specific processes. Summing the three values gives 250 ms for M11 and 189 ms for M12, and subtracting them from the mean experimental RTs of 444/537 ms (C/I, M11) and 492/488 ms (C/I, M12: see Tables 5 and 6), yields the values 195/288 ms (C/I, M11) and 303/299 ms (C/I, M12) for mean decision times predicted by the model. Thus, M12’s decision times and accuracies (≈ 96%) are similar on reponse-hemifield congruent and incongruent trials. M11’s decision process is faster overall, yielding similar accuracy with a mean decision time more than 100 ms *shorter* than M12’s on congruent trials. However, M11’s strong cross-inhibition reduces LIP activity on incongruent trials (Fig 7, row 7), lengthening RTs, and the weak IT-AIP pathway downgrades orientation information, reducing accuracy.

### Model predictions for set size 2 and 6 data

**Fig 9:**
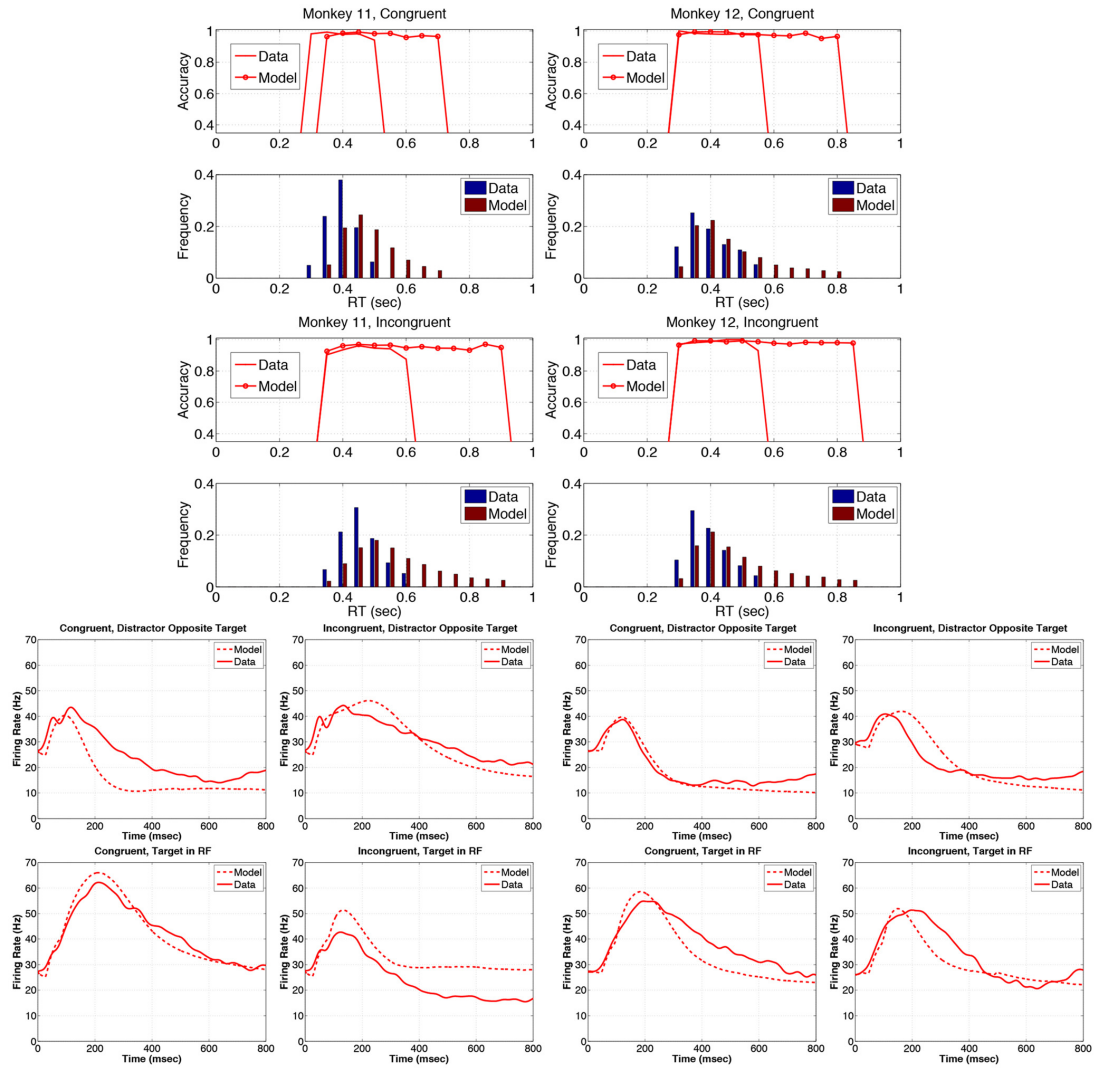
Set size 2 predictions of the model. Using the model parameters fitted to the set size 4 data (Table 3), the input to model was changed to simulate the set size 2 condition. Figure layout analogous to that of Fig 7, but without firing rates for distractor in RF on same side as target, since distractor and target necessarily lie in opposite hemifields for 2 stimuli.

**Fig 10:**
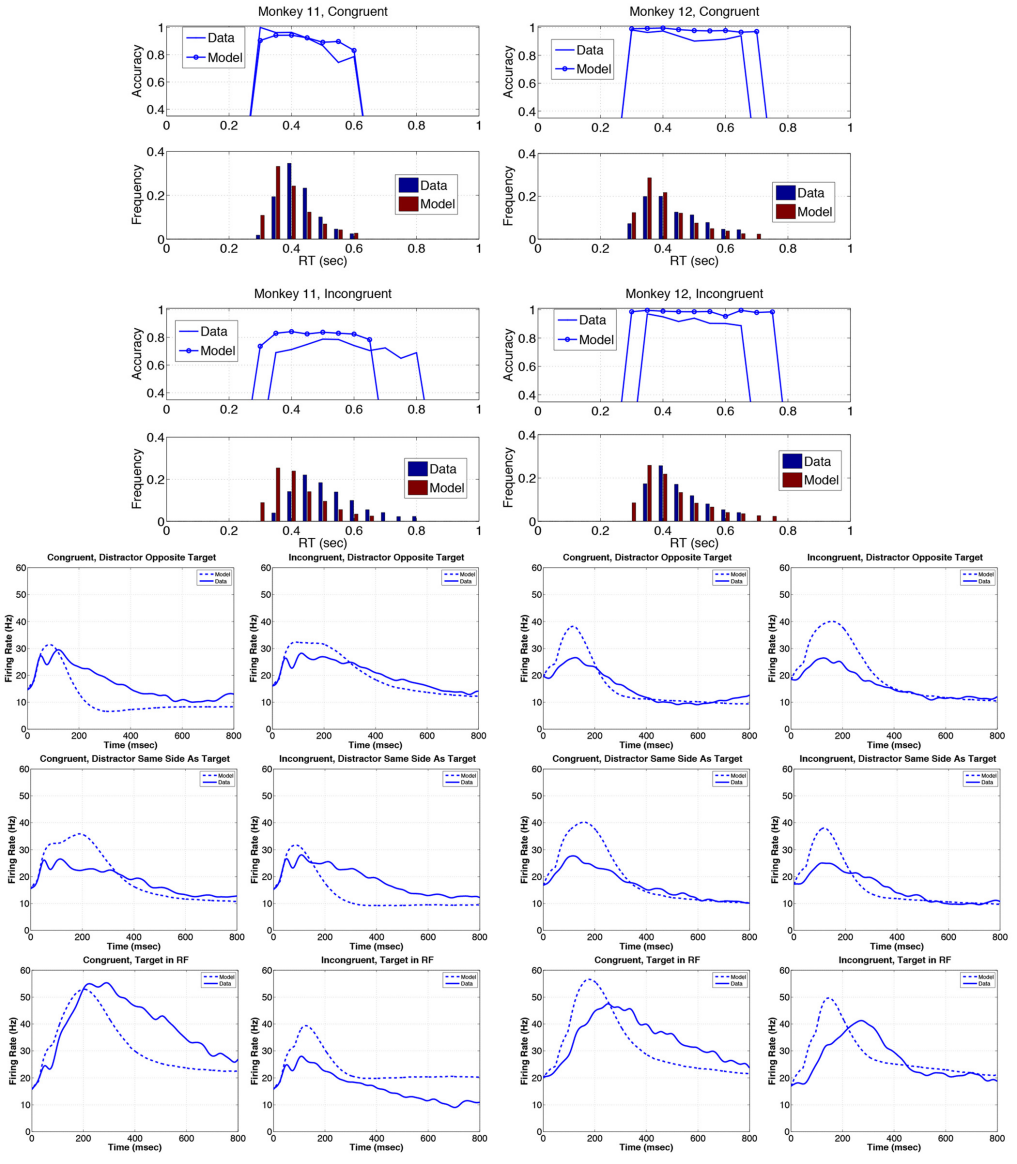
Set size 6 predictions of the model. Using the model parameters fitted to the set size 4 data (Table 3), the input to model was changed to simulate the set size 6 condition. Figure layout analogous to that of Fig 7.

We next ask if the model correctly predicts the set size effect, by simulating the task for set sizes 2 and 6, using the parameter values fitted to each monkey for set size 4 (Table 3). We simply feed the same input signals used above into only 2 or all 6 LIP units. Figs 9–10 show the results.

Set size 2 predictions show some strong qualitative similarities to the data, albeit with notable quantitative deviations. Model accuracies lie within 1.8% of data for both animals and response-hemifield conditions, but the model’s histograms in Fig 9 (rows 1 and 3) show long tails, while the data contain no RTs in these ranges (rows 2 and 4). Unlike set size 4, larger fractions of model trials in these tails (18—28.5%) cause substantial over prediction of mean RTs, which exceed the data by 20% (C and I, M11) and 3.6/11% (C/I, M12). The model underestimates M11’s LIP FRs with distractor in RF on congruent trials and overestimates them with target in RF on incongruent trials, but the other cases for M11 and all cases for M12 are quite well predicted (Fig 9, rows 5 and 6).

Set size 6 predictions of RT distributions and accuracy patterns are generally better than those for set size 2, and much better for congruent trials (Fig 10, rows 1–2). However, the model’s RT distributions are substantially skewed to faster responses for M11’s incongruent trials, accuracy is over predicted by 5.7% and 8.5% for M11 and M12 in this condition (rows 3–4), and M11’s accuracy on congruent trials is over predicted by 5%: see Tables 5 and 6. The model nonetheless captures the decrease in accuracy with RT for M11 although accuracy remains essentially flat for M12. Mean RTs are under predicted by 3.8/15% (C/I, M11) and 13.8/8.4% (C/I, M12). However, LIP FR predictions for M12 are poorer that those for set size 2: peak model FRs are all too high, those for target in RF peak too early and some FRs for M11 decay too slowly. See Fig 10, rows 5–7.

**Fig 11:**
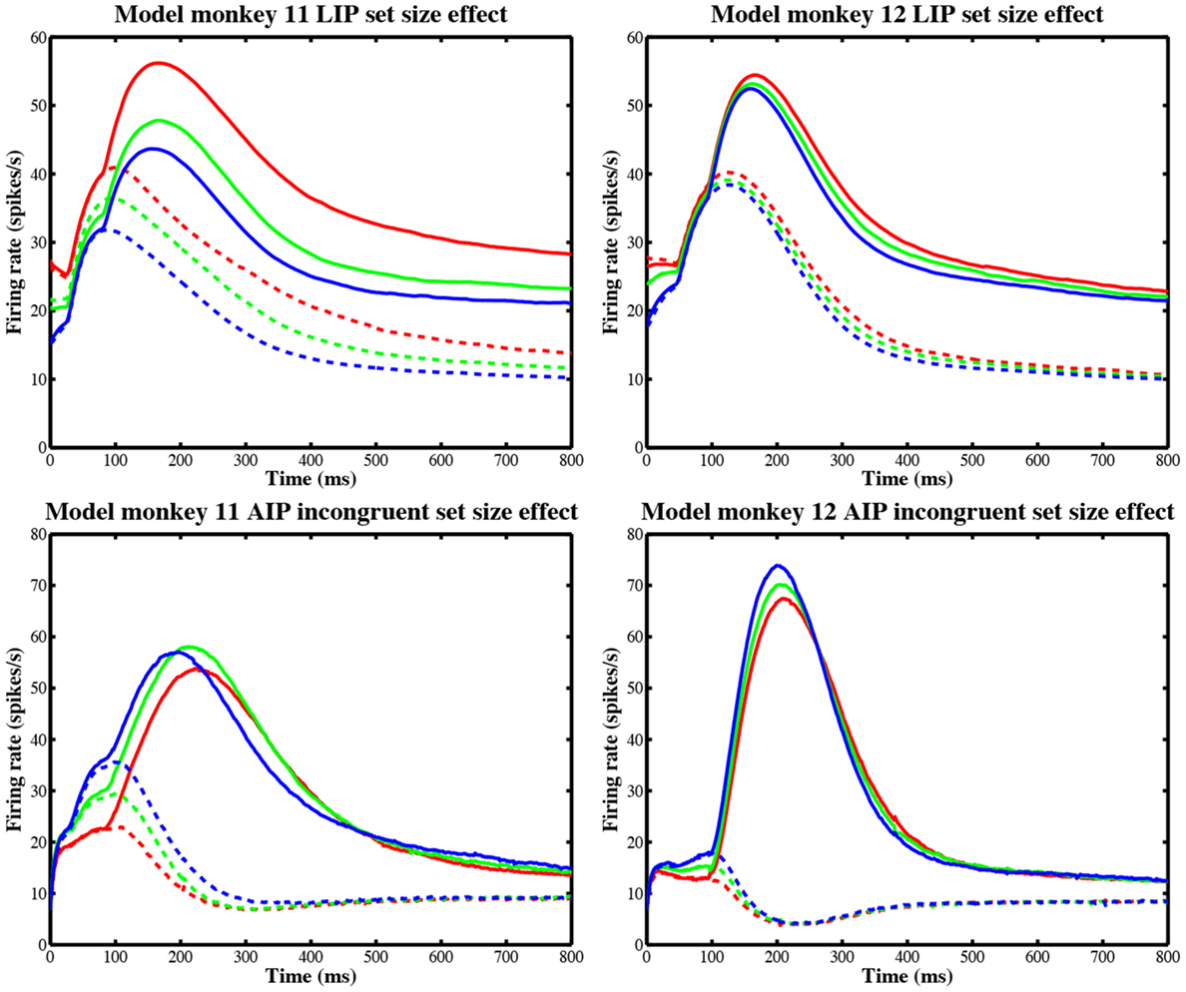
Set size effects on FRs predicted by the model. Model parameters are again given by the fit to the set size 4 data (Table 3). Top row: LIP firing rates (FRs) for monkey 11 (left) and monkey 12 (right) for the different set size conditions: set size 2 (red), set size 4 (green), and set size 6 (blue). Model LIP firing rates (FRs) were averaged over both congruent and incongruent conditions. Also, all LIP units with a distractor in the receptive field (RF) were averaged to produce the distractor FRs (dashed lines). Target in RF is given by the solid lines. Bottom row: FRs in left (solid) and right (dashed) AIP units for a right-facing 𝖤 in the left hemifield, for the incongruent response-hemifield condition only.

These predictions are encouraging, even given the prediction errors for set sizes 2 and 6. The model produces lower accuracies for larger set sizes and captures the qualitative effects of Fig 3, as shown in Fig 11 (top row). As set size increases, LIP FRs for both target and distractor in RF are successively suppressed due to mutual inhibition, although the relative magnitudes for M11 and M12 are reversed (probably due to M12’s much larger LIP inhibition, *η*_*lip*_ = 2.936 compared to 0.1032 for M11) and target and distractor FRs do not coalesce for M11 as quickly in Fig 11 as in the data of Fig 3. Recalling that back inhibition weights from motor units to LIP were constrained to be equal for both animals (Table 3), we conjecture that increasing *β*_*mitlip*_ might improve the collapse of FRs.

The most striking qualitative discrepancy in the model results is the *decrease* in model RTs as set size increases, compared with the increase observed in the data. FRs *of individual LIP units* in the model do decrease with set size (Fig 11 (top row)), but model FRs in AIP modestly *increase* as more LIP units become activated, as shown in Fig 11 (bottom row; for clarity, we show only the incongruent condition). The resulting stronger AIP outputs drive motor units to cross threshold faster for larger set sizes. AIP FRs for M12 are similar in both conditions, but for M11 they peak at 65–75 Hz in the congruent condition (right-facing 𝖤’s in the right hemifield), significantly higher than in Fig 11 (bottom left) and much like M12’s FRs in the incongruent condition. This provides further evidence suggesting M11’s weak IT-AIP connections relative those of M12. The erroneous speed-up in decision time might be corrected by an additional inhibitory mechanism in either the motor or AIP units that modulates their FRs depending on set size. More simply, nondecision delays *T*_*delay*_ could be allowed to depend on set size, and either fit or estimated by incorporating a model for visual search.

The model’s consistent over prediction of accuracy is also notable. Increasing noise levels *c*_*maip*_, *c*_*it*_, *c*_*lip*_ may improve fits. We note that the current fitted values of IT and LIP noise levels *c*_*it*_, *c*_*lip*_ are both higher for M11 than M12 (Table 3), consistent with the animals’ overall accuracies.

The fits presented here were preceded by extensive work in which we fit data from all cells (including the six noted above that were excluded in the fits presented here) with the additional constraint that *P*_*delay*_ and *T*_*delay*_ should coincide for M11 and M12. A brief account of this appears in S1 Text. Using the optimization method described in Materials and Methods, we found parameters close to those described above and distinctions between connection strengths similar to those in Table 3 and Fig 8. This lends some confidence to the dominant pathway interpretation and other results presented above.

## Discussion

We propose a model of a complex decision process based on interactions of brain areas involved in shape discrimination, visual attention and manual action selection (Fig 6), and we quantitatively fit the model parameters to detailed behavioral and electrophysiological data from two macaque monkeys. The model replicates key features of the data, previously described in [14, 15], including the response of neurons in the lateral intraparietal area (LIP) to target location, the effects of the number of visual stimuli (set size) and cue-hemifield congruence on LIP firing rates (FRs) and reaction times (RTs), and a counter-intuitive speed-accuracy trade-off displayed by both animals. In addition, using parameters fitted to set size 4 data, the model fits some but not all of the quantitative effects observed for set sizes 2 and 6 (Figs 7–10 and Tables 4–6).

The LIP responses recorded on this task differed in two important ways from those previously reported on simple decision paradigms. First, rather than increasing up to the time of the final decision as is the case when the decision is reported with a saccade (e.g. [3]), firing rates peaked close to the middle of the reaction time period and declined by the time of the manual response. Second, rather than encoding only visuo-spatial selection as was the case in previous paradigms, LIP firing rates showed additional sensitivity to a non-spatial variable: the limb used to report the shape discrimination. Our model suggests that both of these features reflect interactions between area LIP, which is involved in visuo-spatial selection [34], and skeletomotor areas involved in planning the manual release.

The decline of the LIP response was successfully modeled using an inhibitory signal that arises in the motor area and reduces the responses in both LIP and IT as a motor unit’s FR approaches and crosses threshold. Such a suppressive effect has been proposed as a general-purpose reset mechanism in previous decision-making models [28], and has been observed in EEG event-related potentials, where it was proposed to terminate the allocation of attention [29].

Interactions of visuo-spatial and manual responses, including the congruence effects that differed in the two monkeys, were captured by variability in the connections weights among the three areas, while the intrinsic properties of all areas remained similar for both animals (Table 3). Interestingly, the pronounced asymmetry of incongruent vs. congruent trials found in M11 did not require left-right asymmetry in the LIP-AIP pathways as may be a priori assumed, but emerged from competition between pathways connecting AIP to, respectively, LIP and IT. In M11, connection weights were strong for the excitatory AIP-LIP pathways, which are confined to a single hemisphere and can give rise to a bias toward congruent visuo-manual configurations, and weaker for the IT to AIP pathways, which do not encode information about target location and hence produce no congruent effect. In M12, by contrast, the IT to AIP pathway was dominant, resulting in a much weaker congruence effect. Therefore our findings suggest that differences in long-range connection strengths, whether dictated by anatomy or shaped through each monkeys’ learning of distinct strategies, produced the individual differences that are empirically observed.

As noted in the “Model construction” section of Results, our goal was not to exhaustively account for activity on the task, but to build a model with the simplest architecture that can capture the main effects in the data. It is instructive, however, to consider features that we have deliberately excluded but whose introduction may improve the fits. One such missing feature are feedback projections from the attentional to the feature-selective areas (e.g., LIP to IT), which are supported by studies of the visual and oculomotor systems and thought to mediate attentional effects (e.g. [43, 44, 47, 48]). A second feature is the absence of visual receptive fields in area AIP, which runs contrary to empirical observations (e.g. [49–52]) and may account for the failure of our model to capture the behavioral set-size effects. In its current form the model makes the counterintuitive (and wrong) prediction that RTs will decrease with set size, which may be explained by the fact that the addition of distractors activates more receptive fields in LIP, which in turn converge on a single population of manual response units in AIP and speed up the process of action selection (Figs 9–11). This defect might be corrected by smaller receptive fields in AIP, which may have mutual inhibitory interactions and are topographically connected with those in LIP.

Optimization of parameter sets for the present study took several days on a 24 processor machine, and adding new features will further increase the time required by increasing the search space for the optimal parameter combinations. However, improvements in hardware, coupled with additional software optimization, are likely to render this a feasible goal, and allow us to identify parameters that provide better quantitative fits, provide more precise and confident inferences about differences between the monkeys, and permit analyses of model variants that explore other potential connection architectures within and between areas.

In sum, our work offers an example for multi-area analyses of cognitive processes that are partially constrained by behavioral and electrophysiological data, and could be extended for use with extracellular recordings, local field potentials or imaging data. Their construction could extend our understanding of decision making and its neural substrates to more realistic tasks, which engage multiple brain areas with re-entrant connections and can be solved through alternative, individual strategies.

## Supporting Information

S1 Text Notes on preliminary data fits and modeling. (PDF)

S1 Datasets Electrophysiological and behavioral data from the covert search task [14, 15]. (ZIP)
