# Supplementary material for: A Multi-Area Stochastic Model for a Covert Visual Search Task
Source: PLoS One. 2015 Aug 19;10(8):e0136097. doi: 10.1371/journal.pone.0136097 (PMC4545888; doi:10.1371/journal.pone.0136097)
Supplement: S1 Text — (PDF) [file pone.0136097.s001.pdf]

Supplementary Text

Notes on preliminary data fits and modeling

In earlier attempts to fit the model to set size 4 data we included all the cells and associated behavioral data to maximize statistical power. We also constrained P_{delay} and T_{delay} to coincide for M11 and M12. The resulting connection strengths among LIP, IT and AIP exhibited differences for M11 and M12 much like those in Table 3 in the main text, although values of the internal area parameters $\tau_{lip}, \alpha_{lip}, \eta^2_{lip,decay}, k_m$ and the input strengths s_{lip}, s_{tar} differed more substantially. Specifically, for the all- cells fit with P_{delay} and T_{delay} the same for M11 and M12, $\tau_{lip} = 70.52, \alpha_{lip} = 2.658, \eta^2_{lip,decay} = 0.2501, k_{aip} = 11.17, k_m = 0.00048, s_{lip} = 0.3663$, $s_{tar} = 0.2891$, and $P_{delay} = 22.86$, $T_{delay} = 32.30$, in comparison with the values in Table 3.

Nonetheless, the overall sums of sensori-motor latencies P_{delay} and T_0 and the target search time T_{delay} were 232 and 178 ms respectively for M11 and M12, compared with 250 and 189 ms reported in the Results section (see Table 3). Subtracting 232 and 178 ms from the mean experimental RTs of 444/537 ms and 492/488 ms (Tables 5 and 6 in the main text) yields the values 212/305 ms (C/I, M11) and 314/310 ms (C/I, M12) for mean decision times. These are not too far from the values 195/288 ms (C/I, M11) and 303/299 ms (C/I, M12) obtained in the main text.

Allowing P_{delay} and T_{delay} to vary across M11 and M12 and still including all cells and behavioral data, we obtained fits for which all but 4 parameter values fell within 3% of those in Table 3. The exceptions are $\eta_{maipit} = 5.309$ compared with 4.865, $\eta^2_{lip,decay} = 0.0794$ compared with 0.1300, $\beta_{aiplip} = 3.536$ for M12 compared with 4.266, and $k_m = 0.0168$ compared with $k_m = 0$.

As noted in the main text, the work of [1] showed that significant numbers of LIP neurons exhibit limb preferences, determined by comparing spike counts in a 200 ms window before bar release. If FRs for L and R release trials differed significantly (Wilcoxon rank sum test, $p = .05$), the cell was considered to prefer the limb whose movement yielded greater activity. Cells that failed the test were deemed without limb preference. Preferences were further divided into those favoring the congruent or incongruent limbs. A cell had congruent (C) limb preference if the lateral location of its receptive field (RF) matched the preferred limb (e.g. RF in right visual field and right limb preferred); it had incongruent (I) limb preference if the RF and preferred limb were on opposite sides.

	**Monkey 11**				**Monkey 12**			
	**# cells**	**Non**	**L/R**	**C/I**	**# cells**	**Non**	**L/R**	**C/I**
**Set size 2**	43	5	25/12	36/1	49	22	10/17	14/11
**Set size 4**	32	1	18/13	30/1	43	25	9/9	13/5
**Set size 6**	27	9	14/4	18/0	25	17	4/4	7/1

Table 1. Numbers of neurons with various preferences. First column for each monkey lists the total number of cells available for analysis; second lists number of cells showing no limb preference, others show numbers of cells that preferred left or right limb (L/R) and number of cells that preferred congruent or incongruent limb (C/I).

Table 1 displays the numbers of neurons demonstrating each type of preference, showing that the animals differ substantially: the fraction of non-preference cells for M11 was much less than for M12, and almost all of M11's preference neurons preferred the congruent limb. M12, while also showing a bias for congruence, was overall more "balanced." This led us to consider a model with separate sub-populations of LIP neurons, dividing each RF into three subunits that prefer L, R or neither limb. However, the experimental data lacked sufficient fitting power when split according to Table 1. Moreover, preferences of certain neurons changed with set size, making modeling problematic. Including 18 LIP units would also

incur substantial computational costs, making data fitting even harder. We therefore did not explicitly model neurons with limb preferences.

References

1. Oristaglio J, Schneider DM, Balan PF, Gottlieb J (2006) Integration of visuospatial and effector information during symbolically cued limb movements in monkey lateral intraparietal area. The Journal of Neuroscience 26: 8310-8319.
